# Emergence of immune escape at dominant SARS-CoV-2 killer T cell epitope

**DOI:** 10.1016/j.cell.2022.07.002

**Published:** 2022-08-04

**Authors:** Garry Dolton, Cristina Rius, Md Samiul Hasan, Aaron Wall, Barbara Szomolay, Enas Behiry, Thomas Whalley, Joel Southgate, Anna Fuller, The COVID-19 Genomics UK (COG-UK) consortium, Théo Morin, Katie Topley, Li Rong Tan, Philip J.R. Goulder, Owen B. Spiller, Pierre J. Rizkallah, Lucy C. Jones, Thomas R. Connor, Andrew K. Sewell

**Affiliations:** 1 Division of Infection and Immunity, Cardiff University School of Medicine, CF14 4XN Cardiff, Wales, UK; 2 Systems Immunology Research Institute, Cardiff University, CF14 4XN Cardiff, Wales, UK; 3 School of Biosciences, Cardiff University, CF10 3AX Cardiff, Wales, UK; 4 Department of Paediatrics, University of Oxford, OX3 9DU Oxford, England, UK; 5 Centre for Clinical Research, Royal Glamorgan Hospital, Ynysmaerdy CF72 8XR, UK; 6 Pathogen genomics Unit, Public Health Wales NHS Trust, CF14 4XW Cardiff, Wales, UK

**Keywords:** T cell, T cell receptors, immune escape, SARS-CoV-2, COVID-19, phylogenetic, CD8 T cell, peptide-HLA

## Abstract

We studied the prevalent cytotoxic CD8 T cell response mounted against severe acute respiratory syndrome coronavirus 2 (SARS-CoV-2) Spike glycoprotein_269-277_ epitope (sequence YLQPRTFLL) via the most frequent human leukocyte antigen (HLA) class I worldwide, HLA A^∗^02. The Spike P272L mutation that has arisen in at least 112 different SARS-CoV-2 lineages to date, including in lineages classified as “variants of concern,” was not recognized by the large CD8 T cell response seen across cohorts of HLA A^∗^02^+^ convalescent patients and individuals vaccinated against SARS-CoV-2, despite these responses comprising of over 175 different individual T cell receptors. Viral escape at prevalent T cell epitopes restricted by high frequency HLAs may be particularly problematic when vaccine immunity is focused on a single protein such as SARS-CoV-2 Spike, providing a strong argument for inclusion of multiple viral proteins in next generation vaccines and highlighting the need for monitoring T cell escape in new SARS-CoV-2 variants.

## Highlights

• SARS-CoV-2 Spike mutation P272L emerged and transmitted in several viral lineages
• Residue 272 sits centrally in a frequently targeted, dominant killer T cell epitope
• P272L escaped from >175 T cell receptors raised in cohorts of patients and vaccinees
• Monitoring T cell escape and including further proteins in future vaccines is advised

## Graphical abstract

**Figure:**
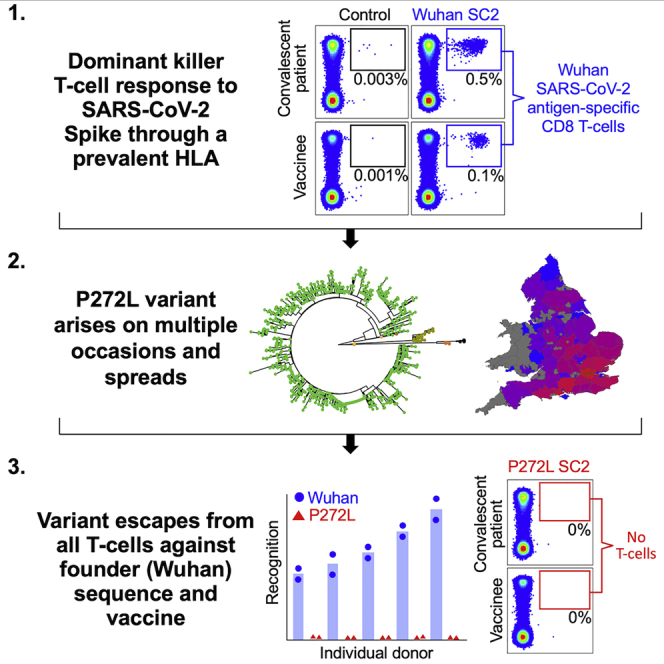

## Teaser

Analysis of the CD8^+^ T cell response to various SARS-CoV-2 lineages identifies a mutation in the viral spike protein that enables the virus to escape T cell responses.

## Introduction

The mammalian immune system utilizes numerous highly developed mechanisms to defend against viral infection. The most sophisticated of these systems, adaptive immunity, is controlled via vast fleets of highly variable antigen-binding molecules called B cell receptors (antibodies) or T cell receptors (TCRs). The advent of SARS-CoV-2 as a novel human coronavirus and the stress on global healthcare systems caused by the associated coronavirus infectious disease 2019 (COVID-19) has focused the world’s attention on ways to combat this emerging infection. The mechanisms used by coronaviruses to escape from the host adaptive immune system are not well understood but are now firmly in the spotlight. The developing picture shows that neutralizing antibodies, CD4 “helper” T cells and CD8 “killer” T cells all contribute to the control of SARS-CoV-2 and the protection offered by currently approved vaccines (Rydyznski Moderbacher et al., 2020; Sette and Crotty, 2021). Although research focused on antibody-mediated immunity has made up the majority of published SARS-CoV-2 immunological work to date, some evidence suggests that antibodies may play a secondary role in ultimately clearing SARS-CoV-2 infection compared to T cells. The presence of SARS-CoV-2-specific CD4 and CD8 T cells has been reported to correlate with reduced COVID-19 severity, while neutralizing antibodies in the same individuals did not (Rydyznski Moderbacher et al., 2020). Two SARS-CoV-2-positive agammaglobulinemia patients who developed COVID-19 symptoms during the first wave of infection in Italy required neither oxygen nor intensive care before making full recoveries (Soresina et al., 2020). The authors conclude that while an antibody-mediated response to SARS-CoV-2 might be important, it is not obligatory for overcoming disease (Soresina et al., 2020). This finding is consistent with multiple patients that developed COVID-19 while on B cell-depleting therapy across several studies, who resolved infection without the need for intensive treatment (Montero-Escribano et al., 2020; Novi et al., 2020; Safavi et al., 2020). There are also many reports of healthy individuals successfully controlling SARS-CoV-2 infection without having detectable neutralizing, or receptor-binding domain (RBD), antibodies while having prominent SARS-CoV-2-specific T cell memory (Nelde et al., 2021; Rydyznski Moderbacher et al., 2020; Schulien et al., 2021; Sekine et al., 2020; Shomuradova et al., 2020). Clinical interventions using monoclonal antibodies further suggest that humoral immunity to SARS-CoV-2, although important, was not the hoped-for panacea for individuals that require intensive treatment (Group et al., 2021; Weinreich et al., 2021).

The association of SARS-CoV-2-specific CD4 and CD8 T cells with milder disease suggests that both T cell subsets play a role in protective immunity (Rydyznski Moderbacher et al., 2020). Indeed, the relative scarcity of naive T cells in individuals over 65 years old and the connection between aging and impaired adaptive immune responses to SARS-CoV-2 has been suggested as a major cause of severe disease (Rydyznski Moderbacher et al., 2020). Analysis of immune cells in bronchoalveolar fluid from patients with COVID-19 showed that moderate disease correlated with highly clonally expanded CD8 T cells (Liao et al., 2020). Acute phase SARS-CoV-2-specific T cells displaying a highly activated cytotoxic phenotype were present in antibody-seronegative exposed family members, indicating that they may be capable of eliminating infection prior to induction of humoral immunity (Sekine et al., 2020), and it has been suggested that strong antibody responses but weak CD8 T cell responses could contribute to acute COVID-19 pathogenesis and severity (Sette and Crotty, 2021; Zhou et al., 2020). Depletion of CD8 T cells in convalescent non-human primates reduced the protective efficacy of natural immunity against SARS-CoV-2 rechallenge (McMahan et al., 2021), suggesting CD8 T cells in the upper respiratory tract may play a similar protective role in humans. Furthermore, vaccination of human angiotensin converting enzyme (ACE)2-transgenic mice with three doses of a peptide incorporating an immunodominant CD8 T cell epitope (Spike_539-546_) protected all animals against lethal SARS-CoV-2 infection in the absence of neutralizing antibodies or CD4 T cells, demonstrating that CD8 T cells alone are sufficient to protect against severe disease (Pardieck, 2022). Given the importance of CD8 T cells to adaptive immune protection against COVID-19, we set out to examine dominant T cell responses to SARS-CoV-2 through the most prevalent major histocompatibility complex (MHC) allele in humans, *HLA A^∗^02* (Gonzalez-Galarza et al., 2020). HLA A^∗^02 is an MHC class I molecule that presents processed intracellular protein antigens at the cell surface in the form of short peptides 8–10 amino acids in length for inspection by CD8 T cells.

SARS CoV-2 infection induces T cells that recognize peptides derived from a range of viral proteins with enrichment for those that respond to Spike, nucleocapsid, membrane, ORF1ab, and ORF3a (Ferretti et al., 2020; Grifoni et al., 2020; Le Bert et al., 2020; Nelde et al., 2021; Sekine et al., 2020). Unbiased screening of nine *HLA A^∗^02*^+^ convalescent patients (CP) showed that the two biggest and most frequent CD8 T cell responses recognized regions of the virus contained within ORF1ab residues 3,881–3,900 and Spike residues 261–280 (Ferretti et al., 2020). The dominant Spike epitope was narrowed down to residues 269–277 (YLQPRTFLL), and T cells that responded to this peptide represented >1 in 10,000 total CD8 T cells in many HLA A^∗^02^+^ CP with the majority of the nine donors using a TCR made with the *TRAV12-1* gene, suggesting a potential shared or “public” response (Ferretti et al., 2020). Another study found that YLQPRTFLL was the most frequently recognized of 13 reported HLA A^∗^02:01-restricted epitopes (responses in 16/17 HLA A^∗^02^+^ CP studied) (Shomuradova et al., 2020). TCR sequencing of HLA A^∗^02:01-YLQPRTFLL tetramer^+^ cells revealed prominent CDR3 motifs that were shared across individuals and confirmed the general *TRAV12-1* dominance (Schulien et al., 2021). TCRβ repertoire analyses also indicated that CD8 T cell responses to the Spike 265–277 region containing the YLQPRTFLL epitope dominate responses to Spike in both CP and individuals vaccinated with “single-shot” Ad26.5.COV2.S vaccine in the US irrespective of HLA type (Alter et al., 2021). We reasoned that if SARS-CoV-2 were to exhibit escape from CD8 T cells, then this would most likely first occur within a dominant T cell response restricted by the most frequent HLA in the population. We therefore focused our attention on residues 269–277 of the SARS-CoV-2 Spike protein and found that the most prevalent mutation in this T cell epitope, YLQ**L**RTFLL (P272L change indicated in bold underlined text), was not recognized by >120 TCRs that responded to the founder epitope (YLQPRTFLL) across a cohort of twelve HLA A^∗^02^+^ CP. We also found sizable populations of CD8 T cells that stained with peptide-HLA A^∗^02:01-YLQPRTFLL multimers in a cohort of individuals that had been vaccinated against SARS-CoV-2. These cells did not stain with reagents manufactured with the P272L variant, recognize exogenous P272L peptide, nor respond to surrogate infected cells expressing Spike protein containing the P272L mutation, suggesting that this variant escapes from vaccine induced T cell responses.

## Results

### Emergence of mutations in the YLQPRTFLL CD8 T cell epitope

**Figure 1:**
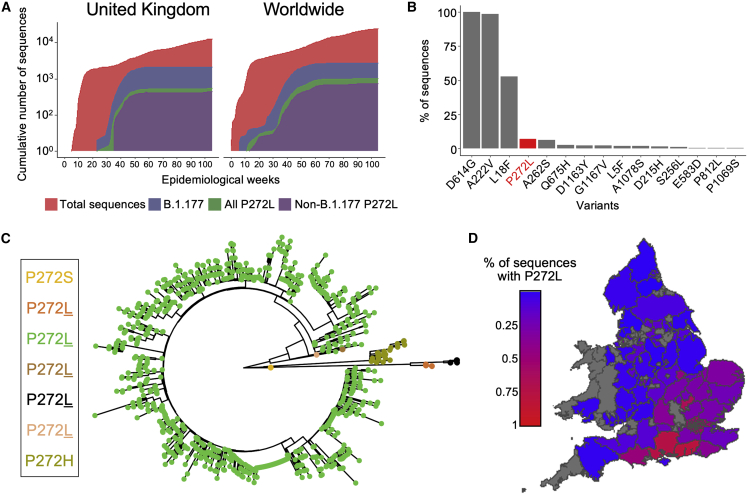
SARS-CoV-2 variation in the immunodominant YLQPRTFLL dominant HLA A^∗^02-restricted CD8 T cell epitope (A) Cumulative frequency of all sequences; sequences in the B.1.177 lineage; and sequences possessing the P272L variant across the United Kingdom (left) and worldwide (right). See Figure S1 for the total number of P272L variants by nation, binned into periods of 16 epidemiological (EPI) weeks is shown. (B) Top 15 most frequently observed variants observed in worldwide Spike glycoprotein sequence data in lineage B.1.177 as a percentage of total sequences. Data for all lineages are shown in Table S1. (C) Phylogenetic tree showing 1,227 taxa with variants at Spike position 272, with colors indicating subtrees representing potential independent mutations, computed by ASR on a larger tree of 200,221 taxa (not shown). (D) Percentage of sequences possessing P272L variant in England and Wales per administrative region up to and including January 1^st^, 2022. See also Figures S1 and S2.

**Figure 2:**
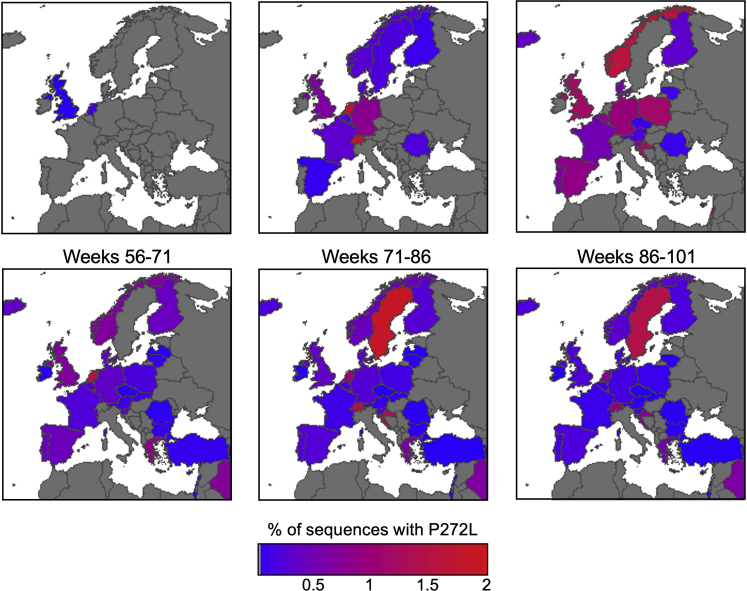
SARS-CoV-2 viral dynamics of P272L Spike mutation in Western Europe Percentage of sequences containing the P272L variant by nation, binned into periods of 16 epidemiological weeks, from week 11 (beginning Sunday, March 8^th^, 2020), the first time a sequence with P272L was observed in sequence data; up to and including November 28^th^, 2021 (week 101). See Figure S1 for worldwide data. See also Figures S1 and S2.

**Figure S1:**
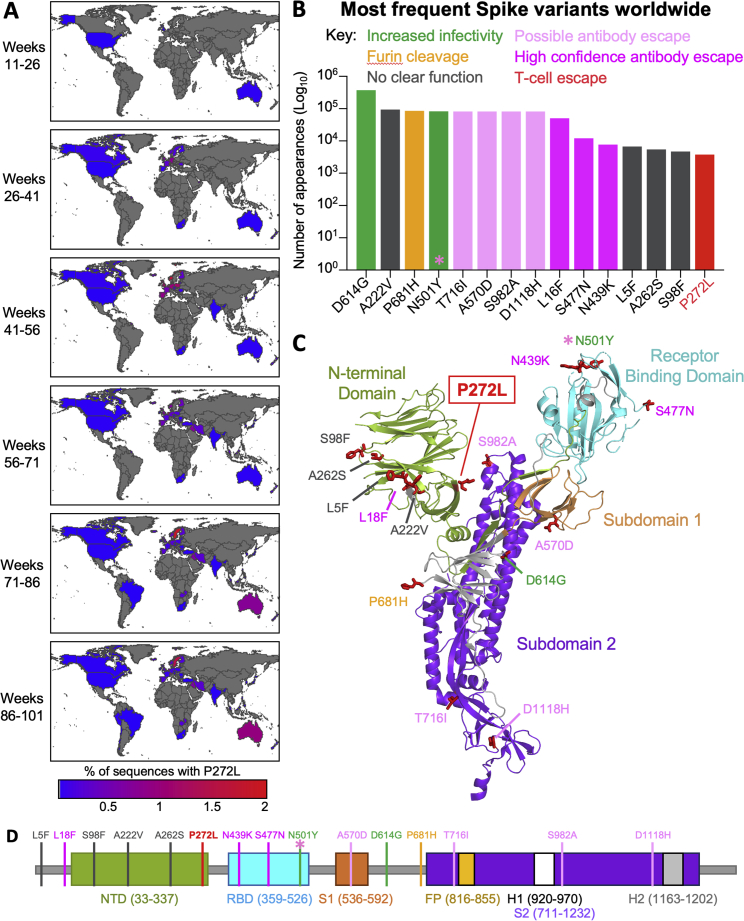
Worldwide SARS-CoV-2 viral dynamics of P272L spike mutation, related to Figures 1 and 2 (A) Percentage of P272L variants by nation, binned into periods of 16 epidemiological weeks, from week 11 (beginning Sunday 8^th^ March 2020), the first time a sequence with P272L was observed in sequence data; up to and including November 28^th^ 2021 (week 101). (B) Top 15 most frequently observed variants observed in worldwide spike glycoprotein sequence data in all lineages up to January 31^st^ 2021. Those thought to be associated with enhanced viral transmission are shown in green and those listed as potentially escaping from antibody mediated-mediated immunity are shown in light pink (possible) and dark pink (high confidence) as assigned at http://sars2.cvr.gla.ac.uk/cog-uk/#shiny-tab-immunology. This colour-coding of substitutions is used throughout the figure. Variants of unknown function in grey. N501Y also escapes from antibodies (pink asterisk). (C and D) Mapping of mutants shown in A onto Spike prefusion structure (PDB 6VXX; (Walls et al., 2020)) and sequence map.

**Figure S2:**
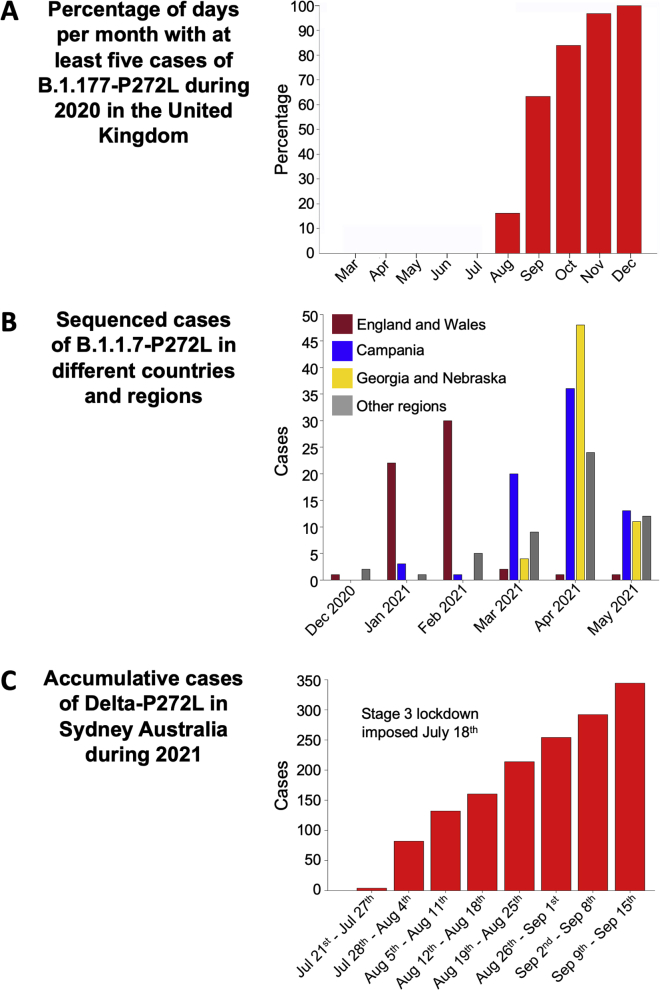
Transmission of P272L variant in B.1.177, B.1.1.7/Alpha and B.1.617.2/Delta variants, related to Figures 1 and 2 (A) Fraction of days per month where five or more P272L variants were sequenced in B.1.177 background. (B) Localised emergence of P272L in the B.1.1.7/Alpha lineage prior to dominance of B.1.617.2/Delta lineage. (C) Cumulative sequenced cases during 2021 in Sydney Australia of P272L mutant in the B.1.617.2/Delta lineage.

The previously unprecedented genome-sequencing efforts for SARS-CoV-2 and sequencing of over 1,600,000 genomes in the United Kingdom to date (>24% of the global effort) has allowed detailed analyses and identification of over a thousand UK transmission lineages, with some variants associating with higher viral loads and increased transmission fitness (du Plessis et al., 2021; Volz et al., 2021). To provide context and examine the extent to which P272L occurs “in the wild,” we examined the global dataset sequenced as of January 1^st^, 2022 and performed focused analyses on the dominant Spike epitope at residues 269–277 (Figures 1, 2**,** and S1), examining mutations that occurred in this region prior to the identification of the first variants of concern, as well as its continued re-emergence over the course of the pandemic to date. Within our dataset, we used ancestral state reconstruction to estimate the number of independent amino acid substitutions that have occurred in between residues 269–277 during the first, second, and third wave in the UK from sequenced data collected from the beginning of the pandemic to January 31^st^, 2022. This analysis identified at least 24 different amino acid changes in this region, 22 of which were seen six or fewer times. The two mutations resulting in the largest number of cases both occurred at position 272 (P272L and P272H). The P272H mutation was basal to a single phylogenetic cluster comprising 26 sequenced cases, while P272L was basal to a single phylogenetic cluster that was internationally distributed and comprised thousands of sequenced cases (Figures 2 and S1). The large, international cluster featured the evolution of P272L on a background of the B.1.177 lineage (Figure 2). B.1.177 is characterized by the lineage-defining Spike mutation A222V and was shown to have arisen in Spain and to have been exported to other European countries and the wider world through travel over the summer of 2020 (Hodcroft et al., 2020). The genomic data indicates that the first sequenced occurrence of P272L in any lineage was identified in March 2020, occurring in lineages B1 and B.1.1.263 before evolving in the B.1.177 background, with the first sequenced case of B.1.177 with P272L being reported in June 2020. Following its emergence, P272L in B.1.177 was sequenced from cases in 29 countries, pointing to extensive spread over the summer and autumn of 2020 (Figures 1D, 2, and S1A). The B.1.177 sub-lineage carrying P272L was likely to have been first imported into the UK in June, shortly after it was first observed (Figure S1A) and spread to become the 4^th^ most frequent Spike variant in B.1.177 in the UK (Figure 1B) before it was displaced by a combination of non-pharmaceutical interventions and by the emergence of the B.1.1.7/Alpha variant. Predominance of the B.1.177 P272L variant was highest in the southeast of the UK and was present in 678/4,317 (∼16%) of all B.1.177 genomes sequenced from Greater London between March 7, 2020 and January 31, 2021. Although B.1.177 was ultimately suppressed and outcompeted by B.1.1.7/Alpha, up until December 2020 B.1.177 with P272L showed growth in multiple parts of the UK (Figures 1D and S2A). While it is not possible to perform a meaningful analysis to examine any selective advantage (the number of samples carrying P272L is not sufficient to detect any signal), in absolute terms P272L was in the top 15 Spike mutations observed globally as of January 31, 2021 (Figure S1B). Moving beyond the first three waves, as of January 2022, P272L has independently emerged, and been sequenced twice or more, in at least 44 lineages other than B.1.177 and its sublineages (Table S1). It is notable that this mutation has arisen spontaneously in variants of concern including B.1.1.7/Alpha, B.1.351/Beta, B.1.617.2/Delta, P.1/Gamma, and BA.1/Omicron, (see Table S1 for details). P272L has also been identified in backgrounds that have been identified over the last year as potential variants of interest/concern including B.1.429 (13 sequenced cases) and B.1.526 (5 sequenced cases). In the case of B.1.1.7/Alpha, P272L was observed in 246 sequenced cases of this lineage as of May 31, 2021 (Figure S2B). 32% (71/220) of reported sequences in the period February–June 2021 were in Campania, Italy (peaking in April 2021) with other local outbreaks of P272L-carrying B.1.1.7/Alpha evident in Nebraska/Georgia USA and the UK (∼29 and ∼14% of total P272L in this lineage February–June 2021). In a similar fashion, the P272L mutation in B.1.617.2/Delta (lineage AY.39.1) resulted in over 300 sequenced samples to date over the course of July–September 2021 in Australia (Figure S2C). Collectively, these data show that P272L has repeatedly emerged in different SARS-CoV-2 lineages, and virus-carrying P272L has gone on to transmit on a local level in multiple countries on multiple occasions.

### The P272L Spike variant is not recognized by YLQPRTFLL-specific CD8 T cells from convalescent patients

**Figure 3:**
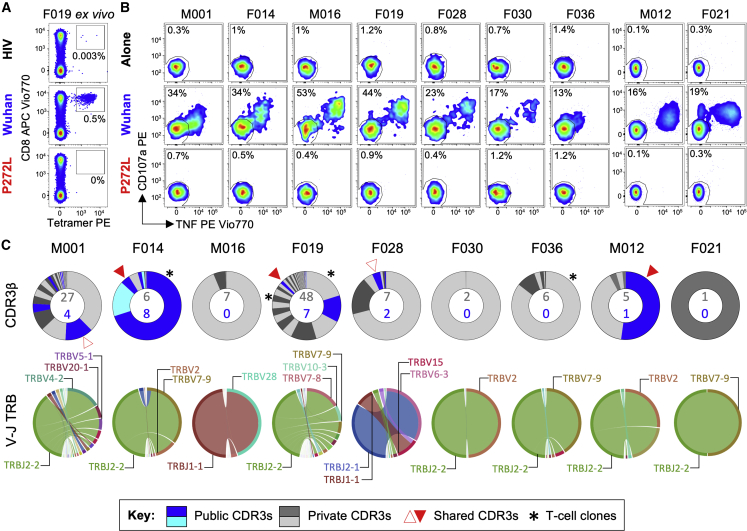
YLQPRTFLL T cell lines from SARS-CoV-2 positive donors do not activate with P272L variant peptide (A) *Ex vivo* PBMC Wuhan-YLQPRTFLL and P272L-YLQLRTFLL tetramer staining of a convalescent patient. HLA A^∗^02:01-SLYNTVATL peptide from HIV used as an irrelevant tetramer (Cole et al., 2017). Percentage of CD8 T cells is shown. *Ex vivo* staining for three other convalescent donors is shown in Figure S3A. (B) Wuhan-YLQPRTFLL T cell lines enriched from nine CPs do not activate towards the P272L-YLQLRTFLL peptide (10^−6^ M used for both peptides). Percentage of reactive cells is displayed from single or duplicate conditions. M012 and F021 analysis performed on a different day. See Figure S3B for a graphical summary of the data. (C) TCR beta chain analysis of Wuhan-YLQPRTFLL tetramer sorted T cells from all patients in A (flow cytometry data in Figure S3E). Pie charts display the proportion (chart segments) and frequency (numbers in center) of public (blue) and private (grey) TCRs. Variable (V) (arc on the right) and joining (J) (arc on the left) gene rearrangements are shown below the pie charts, with the dominant clonotypes annotated. CDR3s sequences are listed in Figure S4A. T cell clones used in this study are indicated with asterisk and data shown in Figure 4. See also Figures S3 and S4.

**Figure S3:**
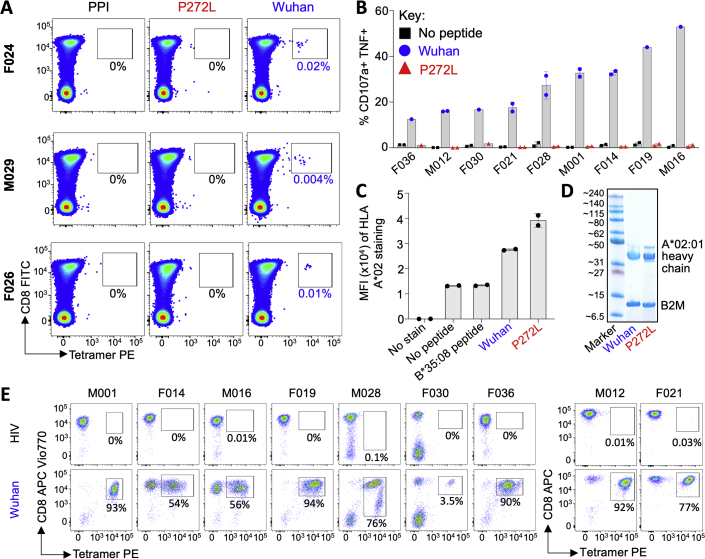
YLQPRTFLL specific T cells from SARS-CoV-2 convalescent patients do not stain or activate with P272L-YLQLRTFLL, yet the variant epitope binds HLA A^∗^02:01 similarly to the Wuhan peptide, related to Figures 3, 4, 5 and 6 (A) PBMCs from three pre-vaccine convalescent patients were stained with HLA A^∗^02:01-Wuhan and P272L tetramers. Preproinsulin (PPI) (ALWGPDPAAA) irrelevant tetramer. (B) PBMCs from convalescent patients were enriched with Wuhan tetramers to create T cell lines, which were used in a 4 h T107a assay with 10^−6^ M Wuhan or P272L peptides. Error bars depict SD of duplicate condititons. Some patient lines performed as a single condition. (C) HLA A^∗^02:01 staining from a T2 cell binding assay using Wuhan and P272L exogenous peptides. HLA B^∗^35:08 binding HPVGEADYFEY was used as an irrelevant control. Error bars depict SD of duplicate conditions. (D) Refolded Wuhan and P272L HLA A^∗^02:01 monomers shown by SDS-PAGE gel used to assemble tetramers for staining T cells. (E) Wuhan tetramer staining of patient T cell lines used for flow cytometry sorting and TCR sequencing. HIV (Gag, SLYNTVATL) tetramers used as a negative control.

**Figure S4:**
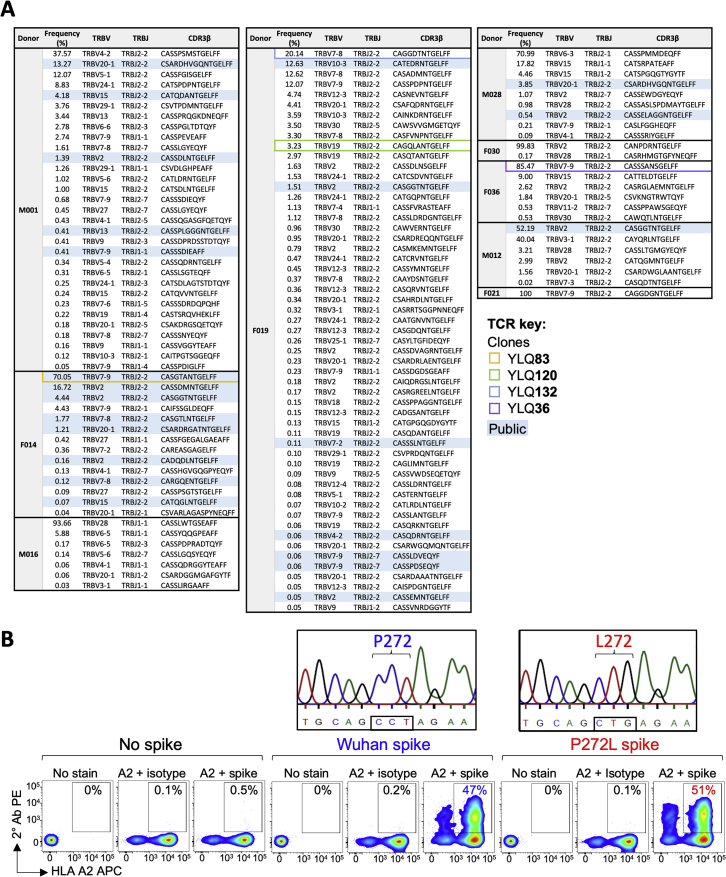
TCR sequencing of Wuhan-YLQPRTFLL tetramer sorted T cells from convalescent patients and Spike transduced A549 cells used to test the reactivity of CD8 T cell clones, related to Figures 3 and 4 (A) TCR sequences of the Wuhan-YLQPRTFLL specific T cells from COVID-19 patients. M012 and F021 analysis performed on a different day. CD8 T cell clones grown from some of the donors annotated according to the key. (B) HLA A^∗^02:01 (A2) and spike protein expression of the Wuhan and P272L spike transduced A549-A2 cells. HLA A^∗^02 staining with BB7.2 Ab clone. Unconjugated isotype and anti-spike antibodies used in conjunction with a PE conjugated secondary antibody on Y axis.

**Figure S5:**
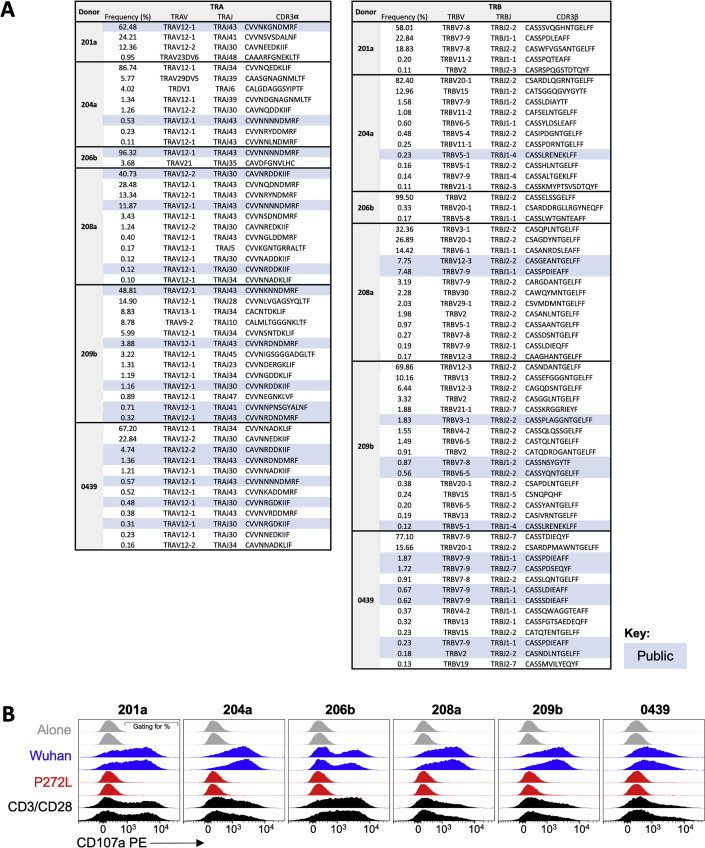
TCRs and peptide reactivity of Wuhan-YLQPRTFLL tetramer enriched T cell lines from six vaccinated donors, related to Figures 5 and 6 (A) TCR usage and CDR3 sequences of Wuhan-YLQPRTFLL specific T cells from vaccinated donors. TCRs that appear in multiple individuals highlighted as public, according to the key. (B) CD107a upregulation of the T cell lines with Wuhan and P272L-YLQLRTFLL peptides. Peptides (10^−6^ M) were pulsed on to T2 antigen-presenting cells prior to assay. CD3/CD28 Dynabeads used as a positive control. Performed in duplicate. Gating of positive cells shown for vaccinee 201a.

**Figure 4:**
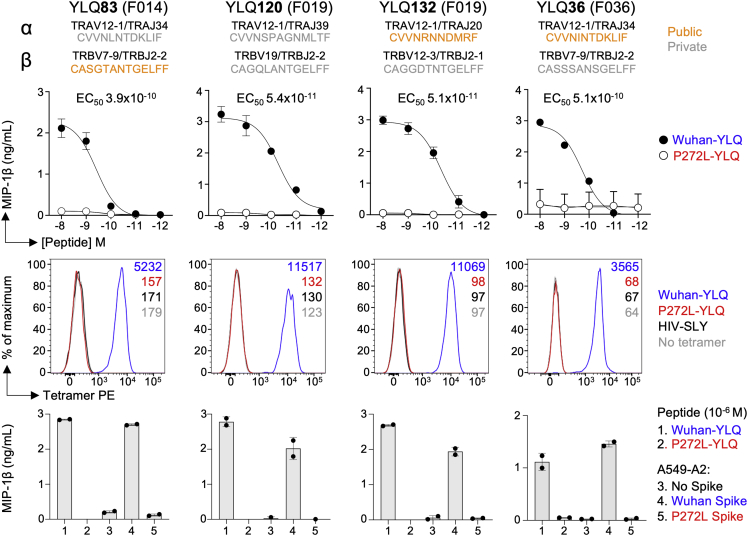
YLQPRTFLL-specific T cell clones from SARS-CoV-2 convalescent patients are unable to recognize P272L YLQPRTFLL peptide reactive CD8 clones were grown from convalescent patients indicated in the brackets. α and β TCR chain usage and CDR3s of each clone are shown and the public or private status of each indicated according to the key. Upper: peptide sensitivity assay with Wuhan-YLQPRTFLL and P272L-YLQLRTFLL peptides. MIP-1β ELISA and error bars depict SD of duplicates. Middle: peptide-HLA tetramer staining. HLA A^∗^02:01 SLYNTVATL (SLY) peptide from HIV used as an irrelevant tetramer (Cole et al., 2017). Lower: A549 cells expressing HLA A^∗^02:01 (A549-A2) and full-length P272L Spike were not recognized, whereas endogenously expressed Wuhan Spike was recognized. MIP-1β ELISA and error bars depict SD of duplicates. See also Figure S4B for HLA A2 and Spike staining of A549 cells. See also Figures S3 and S4.

In order to study the impact of mutation in the YLQPRTFLL epitope, we recruited a cohort of SARS-CoV-2 convalescent local healthcare workers during June 2020. Each had a history of COVID-19 symptoms and a positive nasopharyngeal swab for SARS-CoV-2 by PCR >28 days prior to sample collection. 15/30 donors tested (50%) were HLA A^∗^02^+^ by antibody staining. Twelve of the first thirteen HLA A^∗^02^+^ CP we screened *ex vivo* had CD8 T cells that stained with HLA A^∗^02:01-YLQPRTFLL tetramer (examples in Figures 3A and S3A), confirming the widespread response to this epitope seen in previous studies (Ferretti et al., 2020, Shomuradova et al., 2020). Four of the CP were tested *ex vivo* with YLQ**L**RTFLL tetramer, which failed to stain T cells, despite positive staining with Wuhan tetramers (Figures 3A and S3A). Bulk sorting of tetramer^+^ populations was used to generate a T cell line from nine of the CP. These lines all responded to YLQPRTFLL peptide (13–53% total cells) but completely failed to respond to YLQ**L**RTFLL peptide (Figures 3B and S3B) despite this sequence showing slightly improved binding to HLA A^∗^02:01 compared to the parental (Wuhan) sequence (Figure S3C). The ability of the YLQ**L**RTFLL peptide to bind to HLA A^∗^02:01 was also confirmed during tetramer production (Figure S5D). TCR sequencing of the HLA A^∗^02:01-YLQPRTFLL tetramer^+^ T cells (Figure S3E) in each line identified 128 different TCRs across the nine patients, including the public TCR chains previously identified in addition to many donor-specific TCR sequences (Figures 3C and S4A). Remarkably, these data indicate that all the TCRs failed to respond to the P272L variant sequence. Failure to recognise the P272L mutant or bind to this sequence was confirmed by direct *ex vivo* staining of PBMC (Figures 3A and S3A) and by using four different CP-derived T cell clones (Figure 4). We further confirmed that surrogate infected cells expressing full-length Spike protein with the P272L substitution (Figure S4B) were not recognized by any of these T cell clones (Figure 4). We conclude that P272L has arisen on multiple occasions (appearing to show selection in the B.1.177, B.1.1.7/Alpha, and B.1.617.2/Delta backgrounds) and escapes from all TCRs raised against the parental (Wuhan) sequence that is being used in current vaccines.

### The P272L Spike variant is not recognized by YLQPRTFLL-specific CD8 T cells from vaccinees

**Figure 5:**
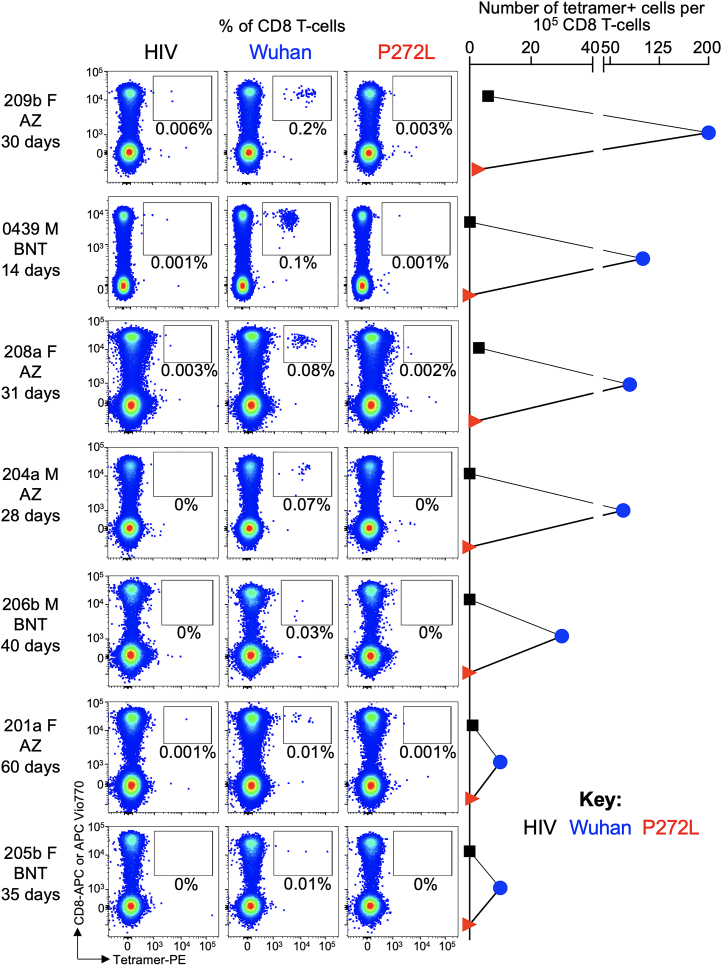
YLQPRTFLL-specific T cells from vaccinees fail to stain with P272L variant tetramers *Ex vivo* tetramer staining of PBMC from 7 vaccinees with HLA A^∗^02:01-YLQPRTFLL and P272L tetramers. Vaccinee gender is indicated next to each donor code. Vaccinees received the AstraZeneca (AZ) ChAdOx1 nCov-19 vaccine or the BNT162b2 (BNT) vaccine as indicated. The number of days post vaccine when blood was taken is indicated for each vaccinee, with data for 0439 shown after the second dose of the vaccine. Tetramer enriched T cell lines were successfully created from 6 of the 7 vaccinees and used for further analysis (Figure 6). See also Figures S3 and S5.

**Figure 6:**
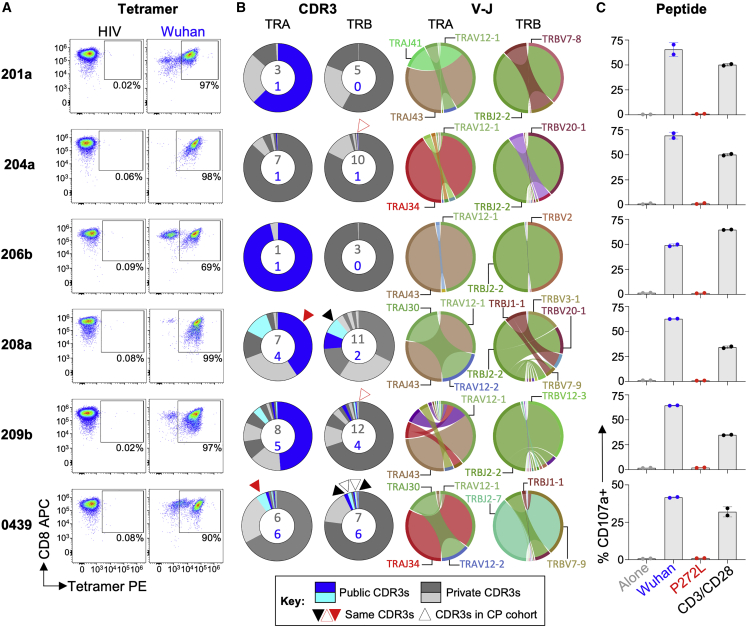
YLQPRTFLL-specific T cell clonotypes from vaccinated donors do not activate with P272L-YLQLRTFLL peptide (A) Wuhan-YLQPRTFLL tetramer enriched T cell lines from 6 of the 7 vaccinees in Figure 5. HLA A^∗^02:01-restricted SLYNTVATL peptide from HIV used as an irrelevant tetramer (Cole et al., 2017). Percentage of CD3^+^ cells is shown. (B) The lines were re-sorted with Wuhan-YLQPRTFLL tetramer for TCR analysis. Pie charts display the proportion (chart segments) and frequency (numbers in center) of public (blue) and private (grey) TCRs. Public or private status based on data from convalescent patient studies. Variable (V) (arc on the right) and joining (J) (arc on the left) gene rearrangements are annotated with dominant and public chains with CDR3s of interest. CDR3 sequences are listed in Figure S5A. (C) CD107a upregulation of the T cell lines with Wuhan-YLQPRTFLL or P272L-YLQLRTFLL peptides. Peptides (10^−6^ M) were pulsed on to T2 antigen-presenting cells prior to assay. CD3/CD28 Dynabeads used as a positive control. Error bars depict SD of duplicates. Flow cytometry data shown in Figure S5B. See also Figures S3 and S5.

We next examined responses to YLQPRTFLL in individuals who had been vaccinated against SARS-CoV-2 and no history of COVID-19 or SARS-CoV-2 infection. In total, PBMC from four HLA A^∗^02^+^ vaccinees who had received a single dose of the AstraZeneca ChAdOx1 nCoV-19 vaccine (Ramasamy et al., 2021) and a further three vaccinees who received the Pfizer-BioNTech BNT162b2 vaccine (Polack et al., 2020) (see materials and methods for donor and vaccination details) were stained with peptide-HLA tetramers. All vaccinees had HLA A^∗^02:01-YLQPRTFLL tetramer staining T cells that accounted for between 0.01% and 0.2% of total CD8 T cells (Figure 5). In all cases, these T cells failed to stain with the HLA A^∗^02:01-YLQ**L**RTFLL (P272L) reagent in parallel assays, indicating that the TCR on these T cells did not strongly engage this sequence in accordance with the CP cohort data (Figure 5). HLA A^∗^02:01-YLQPRTFLL tetramer enriched lines generated from 6 of the 7 vaccinees (Figure 6A) were used for TCR and functional analyses. All six donors had public TCRα CDR3s and a *TRAV12-1* gene bias (37 of 50 chains) as seen in CPs (Figures 6B and S5A). Four of the donors had public TCRβ chains with a total of 58 different TCRs across the six donors (Figures 6B and S5A). None of the vaccinee lines reacted towards exogenous YLQ**L**RTFLL (P272L) peptide, whereas CD107a upregulation ranged between 41%–70% for the YLQPRTFLL peptide (Figures 6C and S5B). We conclude that the YLQPRTFLL-specific T cells induced by two current COVID-19 vaccines fail to functionally engage to the P272L variant sequence.

### Structural comparison of HLA A^∗^02:01-YLQPRTFLL and HLA A^∗^02:01-YLQLRTFLL

**Figure 7:**
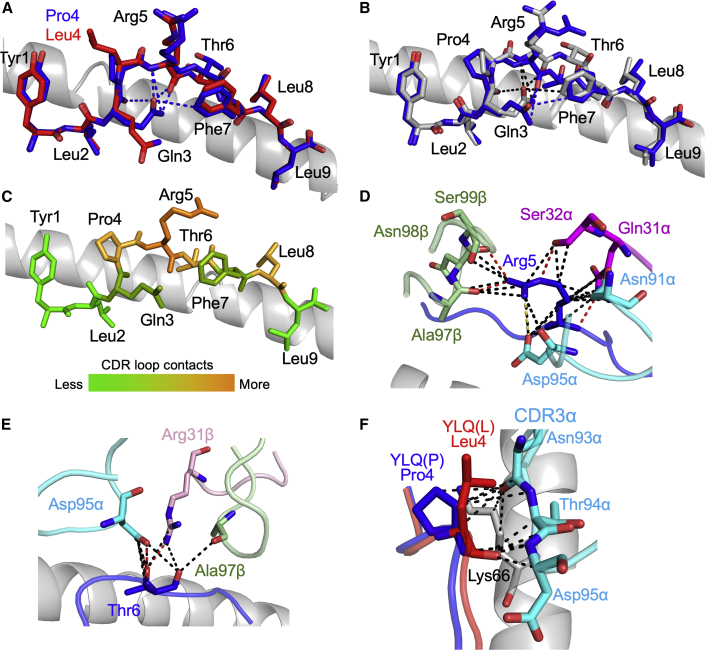
3D structure of antigens and YLQ36 TCR HLA A^∗^02:01-YLQPRTFLL complex (A) Comparison of HLA A^∗^02:01-YLQPRTFLL (blue sticks) and HLA A^∗^02:01-YLQLRTFLL (red sticks). HLA A^∗^02:01 shown as grey cartoon. Intrapeptide bonds present in HLA A^∗^02:01-YLQPRTFLL are shown as blue dashes. (B) Comparison of unbound HLA A^∗^02:01-YLQPRTFLL (grey sticks) and TCR-bound HLA A^∗^02:01-YLQPRTFLL (blue sticks) peptide presentation. HLA A^∗^02:01 shown as grey cartoon. Intrapeptide bonds present in unbound HLA A^∗^02:01-YLQPRTFLL and TCR-bound HLA A^∗^02:01-YLQPRTFLL are shown as black and blue dashes, respectively. See Figure S6 for the sequence of the YLQ36 TCR. (C) Heat map of YLQ36 TCR contacts with the YLQPRTFLL peptide. (D) YLQPRTFLL peptide residue Arg5 shown as blue sticks. Important YLQ36 TCR residues are labeled. Black dotted lines indicate van der Waals interactions. Red dotted lines indicate hydrogen bonds. Yellow dotted lines indicate salt bridges. (E) YLQPRTFLL peptide residue Thr6 shown as blue sticks. Important YLQ36 TCR residues are labeled. Black dotted lines indicate van der Waals interactions. Red dotted lines indicate hydrogen bonds. (F) YLQPRTFLL and YLQLRTFLL P4 residues shown as blue and red sticks, respectively. Important HLA A^∗^02:01 residues shown as grey sticks. YLQ36 CDR3α loop shown as cyan sticks. Interactions involving the YLQ36 CDR3α loop are shown as black dashes. Also see Figure S7. .

T cells are known to be widely crossreactive, allowing some TCRs within polyclonal antigen-specific T cell populations to recognize viral mutations presented at the surface of infected cells in the context of HLA-I molecules (Sewell, 2012). Indeed, individual T cell clonotypes that exhibit greater crossreactivity with known, naturally arising viral variants at an immunodominant CD8 T cell epitope have been suggested to protect against disease progression in HIV infection (Chen et al., 2012). We found it surprising that all 128 HLA A^∗^02:01-YLQPRTFLL-specific TCRs in our CP cohort failed to recognize the P272L variant and that this variant failed to stain or activate T cells bearing >50 TCRs in the vaccinees we studied. To understand why this single amino acid substitution had such a drastic effect on recognition by many TCRs, we solved and compared the structures of YLQPRTFLL and YLQ**L**RTFLL bound to HLA A^∗^02:01 at 1.67 Å and 2.0 Å, respectively (Figure 7A and Table S2). The HLA A^∗^02:01-YLQPRTFLL structure showed the Spike arginine at position 273 (peptide position 5) forms an obvious, protruding, positively charged focal point for TCRs that is stabilized by six intrapeptide bonds including three hydrogen bonds (Figure 7A). The HLA A^∗^02:01-YLQ**L**RTFLL structure shows fewer internal contacts that maintain the shape and dimensions of the bulged part of the epitope (Figure 7A), which may result in the collapse of the peptide bulge upon approach of the YLQ TCR. There is a 2.7 to 3.5 Å further extension of the amino acid 272 side chain in the P272L variant compared with the parent (Wuhan) epitope, suggesting that the longer leucine side chain might interfere with TCR binding (Figure 7A).

### Structure of an HLA A^∗^02:01-YLQPRTFLL-specific TCR

**Figure S6:**
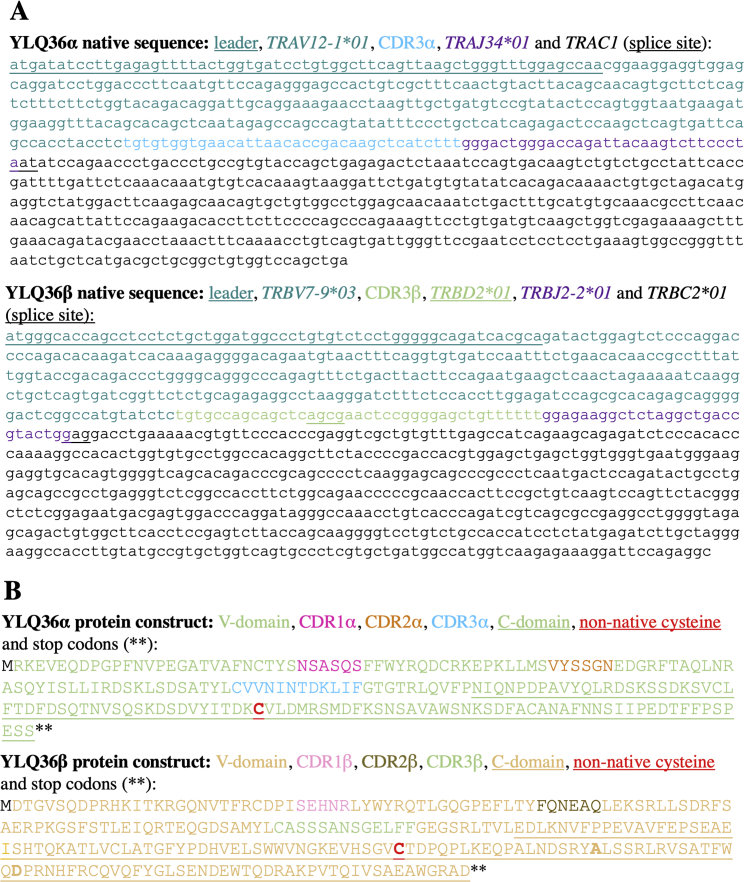
Sequence of the HLA A^∗^02:01 restricted Wuhan-YLQPRTFLL specific YLQ36 TCR, related to Figure 7 and STAR methods (A) Native nucleotide sequence of TCRα and TCRβ genes showing V(D)J assignment. (B) Bacterially expressed protein sequences manufactured in *E*.*coli* and refolded to make soluble YLQ36 TCR for biophysical and structural studies. Sequences include non-native cysteine residues as indicated in bold underlined red text to form non-native disulphide bonding between the TCR α and β constant domains. Two other substitutions in the TCRβ chain that aid refolding are indicated in bold text. The Cys to Ala substitution was included to remove the possibility of incorrect disulphide bind formation.

**Figure S7:**
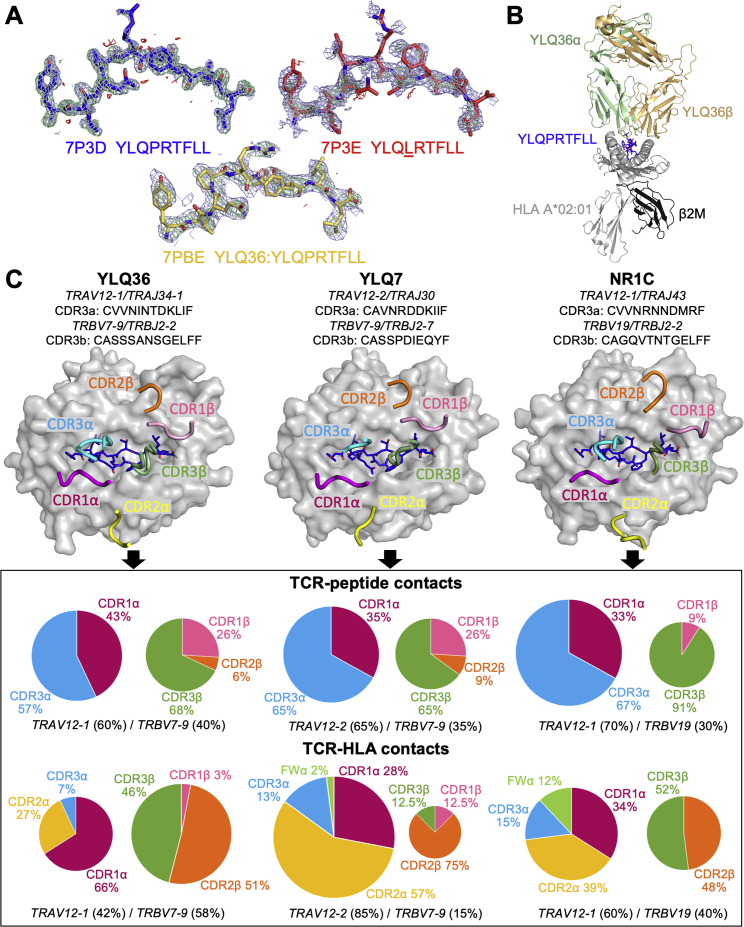
Structural analysis of pHLA and TCRs bound to HLA A^∗^02:01-YLQPRTFLL, related to Figure 7 (A) Omit maps obtained after solving each pHLA structure with PHASER using a model not including the peptide. The density is displayed in light blue from the observed map, green for the positive difference map, and red for the negative difference map. (B) Overview of the YLQ36:HLA A^∗^02:01-YLQPRTFLL 3D structure. (C) TCR footprints: top-down view of YLQ36, YLQ7/YLQ-SG3 (YLQ7 used, PDB:7N1F) and NR1C (PDB:7N6E) TCRs bound to HLA A^∗^02:01-YLQPRTFLL. TCR CDR loops shown as colored cartoon, with the peptide shown as blue sticks. Pie charts beneath each footprint indicate the percentage contribution of each TCR chain, individual CDR loop and framework (FW) residue to the overall contacts within the complexes.

To determine how a T cell could fail to recognize the P272L variant epitope, we solved the 3D structure of the YLQ36:HLA A^∗^02:01-YLQPRTFLL TCR:pMHC complex at 3.0 Å (T cell data in Figure 4 and TCR sequence in Figure S6. Omit maps and crystallographic statistics are present in Figures S7 and Table S2, respectively). The YLQ36 TCR bound in a canonical diagonal orientation (Bridgeman et al., 2012) with the TCRα chain over the N terminus of the YLQPRTFLL peptide and the TCRβ chain at the C-terminus with at crossing angle of 51° (Figure S7). Comparison between YLQPRTFLL peptide presentation in the unbound and bound structures showed the Spike arginine at position 273 undergoes a conformational shift during YLQ36 binding (Figure 7B). Analysis of the contacts between the YLQPRTFLL peptide and the YLQ36 CDR loops during binding showed that Spike Arg273 contributes the most bonds (35%) between the peptide and the TCR, confirming the importance of this residue to the TCR:pMHC interaction (Figures 7C and Table S3). Closer examination of the interactions facilitated by Spike Arg 273 showed the YLQ36 CDR1α, CDR3α, and CDR3β loops all make contacts with this peptide residue (Figure 7D). Further analysis of the TCR:peptide contacts showed that YLQ36 CDR3α loop also makes many contacts with the Spike proline residue at position 272 (Table S3) and the threonine residue at position 274 (Figure 7E), highlighting the crucial contribution of CDR3α to YLQ36:HLA A^∗^02:01-YLQPRTFLL interaction. To assess the potential effect of the P272L mutation on TCR binding, the HLA A^∗^02:01-YLQ**L**RTFLL structure was superimposed onto the YLQ36:HLA A^∗^02:01-YLQPRTFLL using PyMol. The resulting image suggested that the leucine at position 272 in the variant has the potential to protrude within 1 Å of the YLQ36 CDR3α loop, resulting in steric hindrance between the leucine 272 residue and the YLQ36 CDR3α loop (Figure 7F). Based on the structural data presented in this study, we hypothesized that this potential steric hindrance may jeopardize the interactions between the CDR3α and the YLQPRTFLL peptide, resulting in loss of YLQ36 T cell recognition.

Overall, the YLQ36 TCR made 77 contacts with HLA A^∗^02:01-YLQPRTFLL with CDR1α, CDR2α, and CDR3α contributing 26%, 0%, and 34% of the interactions compared to 9%, 2%, and 29% for CDR1β, CDR2β, and CDR3β, respectively (Figure S7). The YLQ36 CDR1α loop contributes more TCR:pMHC contacts than the other germline encoded CDR loops, which is consistent with the observed *TRAV* gene bias seen in TCRs responding to this epitope. As here, previous studies have shown that there is a strong bias for *TRAV12-1* and *TRAV12-2* in HLA A^∗^02:01-YLQPRTFLL-specific TCRs, but not the very similar *TRAV12-3* gene (Ferretti et al., 2020; Shomuradova et al., 2020). *TRAV12-1* and *TRAV12-2* encode for a serine at position 32 in CDR1α, whereas *TRAV12-3* encodes a tyrosine. Ser32 makes 4 van der Waals (VdW) contacts with Gln155 of HLA A^∗^02:01 and 3 VdW contacts and a hydrogen-bond with the Spike Arg273 residue. This bonding network would likely not be possible with the Tyr32 in *TRAV12-3* providing a molecular foundation for the observed V gene bias in HLA A^∗^02:01-YLQPRTFLL-specific T cell populations.

## Discussion

Emerging evidence indicates that HLA-I-restricted CD8 “killer” T cells contribute to the control of SARS-CoV-2 and the immunity to disease offered by the currently approved vaccines (Rydyznski Moderbacher et al., 2020; Sette and Crotty, 2021). Individual HLA-I alleles can be associated with increased likelihood of control or progression of disease with viruses such as HIV (Collins et al., 2020). The immune pressure of prevalent host CD8 T cell responses against HIV results in selection of altered viral sequences and viral immune escape (Leslie et al., 2005; Price et al., 1997, 1998). Influenza virus is also known to gradually escape from CD8 T cells with one study estimating that following the 1968 H3N2 Hong Kong flu pandemic, the virus has lost a prominent CD8 T cell epitope once every three years (Woolthuis et al., 2016). We were interested in whether there was evidence that SARS-CoV-2 might also escape from CD8 T cells. Genome sequencing has shown extensive non-synonymous mutations occurred within the SARS-CoV-2 genome during 2020. Some of these mutations alter viral fitness (Plante et al., 2021), while others have been shown to diminish the binding of antibodies (Harvey et al., 2021). To date, there has been limited study of T cell escape and a broad brush-approach of examining T cell recognition of key transmission variants failed to reveal any evidence that escape was occurring (Tarke et al., 2021). A recent report described a patient on rituximab treatment for non-Hodgkin’s lymphoma who was infected with SARS-CoV-2 for over 300 days without detectable neutralizing antibodies (Stanevich, 2022). During this extended infection period, the virus from this patient formed a single unique clade within the B.1.1 lineage but gained over 30 non-synonymous mutations at a rate that substantially surpassed the evolutionary rate of SARS-CoV-2 in the general population, suggesting prevalent intra-host viral adaptation (Stanevich, 2022). Many of these mutations reduced peptide binding to autologous HLA class I molecules, and two were experimentally validated to escape from the patient’s CD8 T cell response. Another recent report demonstrated that mutations in the RBD that enhance ACE2 binding and viral infectivity concomitantly reduce T cell recognition through HLA^∗^24:02 (Motozono et al., 2021). A further study showed that some SARS-CoV-2 mutations in HLA-I-restricted epitopes resulted in lower binding to the HLA, leading to reduced recognition by CD8 T cells, but there was little evidence of these mutations being disseminated (Agerer et al., 2021). It has been suggested that the L270F (Y**F**QPRTFLL) Spike variant might be an escape mutant (Agerer et al., 2021), but this variant was only seen six times throughout our study period, in six different locations and five different PANGO lineages. From our data, this would suggest that while this mutation does arise, there is no evidence of spread of this variant following sporadic emergence across multiple different SARS-CoV-2 lineages. A further study recently demonstrated the potential of SARS-CoV-2 mutations to escape from CD8 T cell responses to ORF3a and nucleocapsid (de Silva et al., 2021). Collectively, the above evidence demonstrates that SARS-CoV-2 can alter its sequence to escape from CD8 T cells within a given host, but there has been no proof of transmission of T cell escape variants (Tarke et al., 2021). We set out to look for evidence for dissemination of CD8 T cell escape in the wild.

We reasoned that if escape variants were to disseminate, then they would be most likely to first be seen in a prevalent response through a frequent HLA. HLA A^∗^02 is frequently carried by all human populations except those of recent African ancestry, where it is present at lower levels. HLA A^∗^02 is believed to be the most frequent HLA-I in the human population worldwide, occurring in ∼40% of individuals (Gonzalez-Galarza et al., 2020). HLA A^∗^02 is hypothesized to have become so frequent in the population due to its success at presenting well-recognized epitopes from historically dangerous pathogens, a supposition consistent with this HLA having entered the population via interbreeding with Neanderthals and Denisovans after early humans migrated from Africa (Abi-Rached et al., 2011). A previous study used overlapping peptides from the entire SARS-CoV-2 proteome to identify that the most common HLA A^∗^02-restricted responses in CP were to epitopes contained within peptides spanning residues 3,881–3,900 of ORF1ab and 261–280 of Spike (Ferretti et al., 2020). The Spike epitope was narrowed down to residues 269–277, sequence YLQPRTFLL. A further study confirmed the prevalence of CD8 T cells that respond to YLQPRTFLL in CP that are absent in healthy donor blood taken before the spread of SARS-CoV-2 (Shomuradova et al., 2020). We confirmed the prevalence of responses to this epitope in our cohort where responses were observed in 12/13 HLA A^∗^02^+^ CP tested and all 7/7 SARS-CoV-2 vaccinees, including those who had received only the first dose of a double dose schedule.

We focused our attention on nonsynonymous mutations in the sequence encoding YLQPRTFLL. While at least 73 different amino acid changes have occurred in this region, 33 were seen in more than six sequenced cases in the period from the beginning of the pandemic to January 1^st^, 2022. The occurrences of mutations in this region that were basal to phylogenetically grouped clusters of 100+ cases all occurred at position 272 with the P272L mutation occurring in over 10,500 sequenced cases as of January 1^st^, 2022. Over the course of the pandemic to date, the P272L variant has arisen independently in at least 10 different PANGO lineages during the study period but was most prevalent in the B.1.177 lineage that played a key role in establishing the “second wave” of SARS-CoV-2 in the UK and Europe during the autumn of 2020. P272L was the 4^th^ most common variant seen in the B.1.177 lineage and its descendants and in the top 15 Spike mutations observed globally to January 31^st^, 2021. Twelve of thirteen of our HLA A^∗^02^+^ CP cohort tested made a response to the YLQPRTFLL epitope, a response comprising >120 different TCRs in total for nine of the patients. Remarkably, no response was seen to the P272L variant. This finding was confirmed using four CP-derived T cell clones that included TCR α or β chains that have previously been described in other CP cohorts by independent research groups (Ferretti et al., 2020; Shomuradova et al., 2020). Responses to the YLQPRTFLL epitope in a cohort of HLA A^∗^02^+^ healthy donors who had been vaccinated against SARS-CoV-2 but had never had symptoms or a positive test since January 2020 ranged from 0.01%–0.2% of CD8 T cells by peptide-HLA staining. However, all of the T cells raised against the YLQPRTFLL epitope across our vaccinee cohort (>50 TCRs) engaged the P272L very poorly and did not stain with P272L peptide-HLA tetramers or react to P272L peptide. The fact that >175 different TCRs from CP and vaccinees raised against the YLQPRTFLL epitope all fail to see the P272L variant suggests that either the proline at position 272 must form a major focus of TCR contact that a leucine residue cannot compensate for, or that substitution with leucine at this position must interfere with a commonly shared TCR binding mode (or both).

Comparison of the atomic structures of HLA A^∗^02:01-YLQPRTFLL and HLA A^∗^02:01-YLQ**L**RTFLL showed a largely preserved fold, but with local geometry alterations at the epitope residues 3 to 5 (Spike residues 271–273) which presumably form the majority of contacts with TCRs. In order to study the interaction between a TCR and HLA A^∗^02:01-YLQPRTFLL, we attempted to make soluble forms of the TCRs from the four T cell clones we generated from CP. We were successful in manufacturing and crystallizing the YLQ36 TCR in complex with HLA A^∗^02:01-YLQPRTFLL. The structure of this complex at 3.0 Å showed a canonical mode of TCR binding with a crossing angle of 51°. As the YLQ36 T cell failed to recognize the P272L variant or stain with P272L tetramers, we examined the effect substitution of proline 272 with leucine might have by superposing the P272L variant peptide (PDB 7P3E) into the TCR complex with the Wuhan epitope. This model showed that the leucine at position 272 in the variant would protrude within 1 Å of the YLQ36 CDR3α loop that forms many important contacts with both peptide and HLA. This would be much closer than the allowed VdW contact distance and would result in repulsive forces. According to Figure 7F, accommodation of the P272L variant by the YLQ36 TCR would require a movement of the CDR3α loop of at least 1.3 Å, lengthening or breaking >25 molecular bonds with the peptide and 4 bonds with the MHC, to potentially result in a substantial loss in binding affinity. However, the P272L mutant side chain may also adopt a rotamer that would render it clear of the TCR if no other conformational changes occur, though it is unclear whether such rotamers would be energetically favorable. A comparison of the Wuhan and P272L variant peptides may also suggest why the YLQ36 T cell does not recognize the P272L variant. Structural analysis of HLA A^∗^02:01-YLQ**L**RTFLL indicates there is a lack of intra-peptide bonds present in the P272L peptide compared to HLA A^∗^02:01-YLQPRTFLL Figure 7A. This may be due to the greater flexibility of leucine compared to proline, which would allow a reduction in the distance between the Gln271 and Arg273 Cα atoms, resulting in potential clashes between the Gln271 side chain and Gln271/Arg273 main chain, which would be relieved by breaking any bonds formed. The lack of support in the P272L variant peptide resulting from a lack of intra-peptide bonds may cause the peptide to collapse under YLQ36 TCR approach, displacing the peptide and abolishing peptide:TCR interactions. While our structural data do suggest steric interference may be responsible for the lack of P272L variant recognition (Figure 7F), other factors may also be responsible for this loss of recognition, which would require further study to dissect.

Three other TCR complexes with HLA A^∗^02:01-YLQPRTFLL have been generated in recent months (Chaurasia et al., 2021; Szeto et al., 2021; Wu et al., 2022), albeit that two of these studies examined the same TCR (Szeto et al., 2021; Wu et al., 2022). The three unique TCRs, YLQ36, NR1C (PDB:7N6E), and YLQ7/YLQ-SG3 (PDB:7N1F/PDB:7RTR), all adopt roughly the same footprint and binding strategy atop HLA A^∗^02:01-YLQPRTFLL where TCR contacts with peptide are dominated by CDR3α with a strong supporting role from the germline-encoded CDR1α loop (Figure S7). The NR1C TCR is comprised of *TRAV12-1/TRAJ43* CVVNRNNDMRF and *TRBV19/TRBJ2-2* CAGQVTNTGELFF chains and binds to HLA A^∗^02:01-YLQPRTFLL with a K_D_ of 2.71 μM (Chaurasia et al., 2021). Mariuzza and colleagues generated the YLQ7 TCR by pairing TCR α and β chains seen in HLA A^∗^02:01-YLQPRTFLL tetramer^+^ CD8 T cells in CPs. While our YLQ36 and the NR1C TCRs use the most common *TRAV12-1* V gene seen in HLA A^∗^02:01-YLQPRTFLL-specific T cells, YLQ7 uses *TRAV12-2* (*TRAV12-2/TRAJ30* CAVNRDDKIIF; *TRBV7-9/TRBJ2-7* CASSPDIEQYF) (Wu et al., 2022). YLQ36 *TRAV12-1*-encoded CDR1α Ser32 makes 4 contacts with Gln155 of HLA A^∗^02:01 and 4 contacts with Spike Arg273, including a hydrogen bond. The CDR1α Ser32 of the NR1C TCR makes 2 contacts with Gln155 (both of which are hydrogen bonds), and the *TRAV12-2*-encoded CDR1α Ser32 in the YLQ7/YLQ-SG3 TCR makes 3 contacts with Gln155 of HLA A^∗^02:01, including 1 hydrogen bond, and 2 contacts with Spike Arg273. The common binding mode of these TCRs (Figure S7) and importance of Ser32 provides a molecular explanation for the observed V gene bias towards *TRAV12-1* and *TRAV12-2* seen in HLA A^∗^02:01-YLQPRTFLL-specific T cell populations in CPs and vaccinees.

The NRIC TCR was shown to bind to HLA A^∗^02:01-YLQPRTFLL with a K_D_ of 2.71 μM, very similar to that observed with three other HLA A^∗^02:01-YLQPRTFLL-specific TCRs in the same study (Chaurasia et al., 2021). The *TRAV12-2* YLQ7/YLQ-SG3 TCR was also included in this study but named NR1F. The three studies that included the YLQ7/YLQ-SG3 TCR reported that it bound to HLA A^∗^02:01-YLQPRTFLL with K_D_ 1.8–6.6 μM (Chaurasia et al., 2021; Szeto et al., 2021; Wu et al., 2022). These results are of the same order of magnitude and fall firmly within the normal range seen for most interaction with viral peptides (Dolton et al., 2018). The four TCRs examined by Chaurasia et al. bound to the HLA A^∗^02:01-YLQ**L**RTFLL variant antigen with an average of >68-fold weaker affinity than to the Wuhan HLA A^∗^02:01-YLQPRTFLL antigen in parallel experiments (Chaurasia et al., 2021). These findings are in accordance with those of Mariuzza and colleagues who showed that the P272L variant antigen bound to the YLQ7 TCR with >70-fold lower affinity and are consistent with the potential structural rearrangements required to accommodate the longer leucine residue at position 272. Such large reductions in TCR binding are expected to drastically reduce T cell activation and are consistent with our finding that HLA A^∗^02:01-YLQPRTFLL-specific T cells from CP or vaccinees fail to respond to physiological levels of the P272L variant.

SARS-CoV-2 variants have been rapidly emerging throughout the current pandemic with mutants that enhance transmissibility, evade host immunity, or increase disease severity being of particular concern (Tao et al., 2021). Current systems for identifying variants of public health concern involve risk assessments that identify mutations that are present, and then look for mutations that have a known biological effect in order to assess their significance. While extensive amounts of work have been undertaken to predict and characterize the likely effects of mutations in Spike on antibody recognition, the same is not true for T cells. Current risk assessments that only consider antibody affinity and factors associated with transmissibility (e.g. mutations that improve cellular infectivity) are clearly incomplete. The range of potential effects of mutations that affect recognition by T cells is significant and urgently requires further study. It is clear that there was a significant SARS-CoV-2 outbreak that spanned Europe of a lineage carrying a mutation that we have shown has a detectable impact on T cell recognition, which went unrecognized at the time. Ultimately, it is unclear whether SARS-CoV-2 incorporates enough genetic plasticity to allow it to escape from humoral immune responses. However, the area targeted on the RBD by neutralizing antibodies is large enough, and the range of epitopes targeted in polyclonal human sera broad enough, to ensure that no single mutation should allow complete escape from neutralization in the majority of individuals (Rees-Spear et al., 2021). Likewise, the wide array of HLA across the population (Gonzalez-Galarza et al., 2020) and the broad range of epitopes responded to in COVID-19 patients (Grifoni et al., 2020) combine to make it unlikely that SARS-CoV-2 will completely escape from T cell surveillance in the near future. Over the course of the pandemic to date, it is increasingly clear that mutations that enable the adaptation of SARS-CoV-2 to bind to the human ACE2 receptor or gain entry into human cells have been strongly selected for, with the virus exhibiting significant jumps in fitness with the evolution of each new variant of concern. While a range of mutations conferring resistance to antibody binding to specific parts of the Spike protein have been identified (e.g. E484K), there has been little evidence to date of mutations that may confer an advantage at an epitope targeted by T cells being selected for on a population-wide level. Examining our data, it is clear that P272L in B.1.177 was on an upward trajectory in the autumn of 2020, but this lineage was ultimately displaced by B.1.1.7/Alpha, which had a much higher growth advantage due to a range of mutations selecting for cell entry and ACE2 binding. It is important to note that although P272L in B.1.177 was clearly increasing in frequency in late 2020, the case numbers concerned, despite being relatively large, would not be sufficient to detect a signal of a growth advantage compared to B.1.177 lineages lacking the P272L variant at the time. When combined with the analysis of the impact of the P272L change characterized here, it is likely that P272L conferred some advantage, but it was also clear that at the time that advantage was not on the same scale as the selective advantage that was conferred by mutations increasing transmissibility in B.1.1.7/Alpha.

It is important to note, however, that as the pandemic progresses, and SARS-CoV-2 reaches an optimal position with regards to ACE2 binding and cell entry, it is likely that other selective advantages (such as T cell escape) will become more important in the mix of potential advantages that the virus could develop to enable it to persist as a human pathogen. It is also instructive that P272L has continued to emerge on a local level since its first emergence in early 2020, including in all variants of concern identified to date. Amongst these, P272L emerged and showed local spread in both B.1.1.7/Alpha and B.1.617.2/Delta, including significant numbers of cases in Australia, Italy, and the US. Based upon these observations, we believe that P272L, and other similar mutations that will impact T cell epitopes, will become increasingly important as a route for SARS-CoV-2 lineages to increase their competitiveness, particularly as global vaccination rates increase. Our data suggest that the 269–277 epitope of Spike is one region that should be monitored, and its impact considered as part of the development of next generation vaccines. Furthermore, the potential of T cell escape presented by mutations in this region would argue for the inclusion of mutations in the 269–277 epitope of Spike in risk assessments by public health agencies going forward.

In summary, we demonstrate that SARS-CoV-2 can readily alter its Spike protein via a single amino acid substitution so that it is not recognized by CD8 T cells targeting the most prevalent epitope in Spike restricted by the most common HLA-I across the population. While it is not possible to directly attribute the emergence and propagation of the Spike P272L SARS-CoV-2 variant in parts of the world where *HLA A^∗^02*:*01* is frequently expressed to CD8 T cell-mediated selection pressure, specific focusing of immune protection on a single protein (e.g. SARS-CoV-2 Spike favored by all currently approved vaccines [Krammer, 2020]) is likely to enhance any tendency for escape at predominant T cell epitopes like YLQPRTFLL. Our demonstration that mutations that evade immunodominant T cell responses through population-frequent HLA can readily arise and disseminate, strongly suggests that it will be prudent to monitor such occurrences and to increase the breadth of next generation SARS-CoV-2 vaccines to incorporate other viral proteins.

### Limitations of the study

A major limitation of this study is that due to the nature of our samples, we were unable to demonstrate that the P272L mutation arose in individuals who were HLA A^∗^02^+^. The nature of the available sequencing data also makes it impossible to track specific individuals in terms of who infected whom, and we were unable to determine whether individuals infected with the P272L variant had a higher viral load than those infected with the same virus without the mutation. Virus from less than 1% of infected individuals was sequenced so the P272L variant outbreaks we report on are likely underestimated in number by at least two orders of magnitude. It is also impossible to definitively attribute the P272L mutation to T cell escape. Furthermore, each time a more infectious variant has replaced the dominant virus worldwide as the pandemic virus shifted from Wuhan to B.1.177 to B.1.1.7/Alpha to B.1.617.2/Delta to B.1.1.529/Omicron, the clock has been reset in terms of the P272L mutation. Viral variation that enhances transmission will almost certainly outcompete immune escape variants during the early stage of a pandemic. Indeed, studies of influenza virus following the 1968H3N2 pandemic show that the virus has lost a predominant CD8 T cell epitope once every three years up to 2015 (Woolthuis et al., 2016). With SARS-CoV-2, this process can only really begin in earnest once traits that enhance transmission have been optimized, a process that is clearly still ongoing. Consequently, our study is limited by being too early after the emergence of SARS-CoV-2 as a human coronavirus to expect to observe any “pure” T cell escape to fixation. A further limitation is whether previously infected or vaccinated individuals can raise a *de novo* response to the P272L variant. It is possible that the YLQ**L**RTFLL sequence falls into a naïve TCR repertoire “blind spot” that most individuals cannot recognize, or that original antigenic sin (Klenerman and Zinkernagel, 1998) maintains the response to 272P Wuhan sequence in the face of the variant. These questions were beyond the scope of the current study.

## STAR★Methods

### Key resources table

**Table:** 

REAGENT or RESOURCE	SOURCE	IDENTIFIER
**Antibodies**		

Mouse anti-human HLA A2 FITC (Clone BB7.2)	BioLegend UK Ltd	Cat# MCA2090F; RRID: AB_324186
Mouse anti-human CD3 PerCP (Clone BW264/56)	Miltenyi Biotec	Cat# 130-113-131; RRID: AB_2725959
REAffinity anti-human CD8 APC (Clone REA734)	Miltenyi Biotec	Cat# 130-110-679; RRID: AB_2659237
Mouse anti-human CD8 APC Vio770 (Clone BW135/80)	Miltenyi Biotec	Cat# 130-113-155; RRID: AB_2725983
Mouse anti-human CD8 FITC (Clone BW135/80)	Miltenyi Biotec	Cat# 130-080-601; RRID: AB_244336
TNFα PE-Vio770, anti-human (clone cA2)	Miltenyi Biotec	Cat# 130-120-492; RRID: AB_2784483
Mouse anti-human CD107a (LAMP-1)-PE, (H4A3)	BD Biosciences	Cat# 555801; RRID: AB_396135
Mouse anti-human CD14 Pacific Blue™ antibody (Clone ME52)	BioLegend UK Ltd	Cat# 301828; RRID: AB_2275670
Mouse anti-human CD19 Pacific Blue™ antibody (clone HIB19)	BioLegend UK Ltd	Cat# 302232; RRID: AB_2073118
Purified mouse anti-PE antibody (Clone PE001)	BioLegend UK Ltd	Cat# 408102; RRID: AB_2168924
Mouse anti-rat CD2 antibody (clone OX-34)	BioLegend UK Ltd	Cat# 201303; RRID: AB_2228899
Unconjugated mouse anti-SARS Spike glycoprotein antibody (clone 1A9)	Abcam	Cat# ab273433, RRID: AB_2891068
Goat Anti-Mouse Ig PE (polyclonal secondary)	BD Biosciences	Cat# 550589, RRID: AB_393768
Mouse IgG1 isotype (P3.6.2.8.1)	eBiosciences, Thermo Fisher Scientific	Cat# 14-4714-82 RRID: AB_470111

**Bacterial and virus strains**		

Competent BL21 DE3 *Escherichia coli* cells	Thermo Fisher Scientific	Cat# C600003
XL10-Gold Ultracompetent cells	Agilent Technologies	Cat# 200315

**Biological samples**		

Blood samples from healthy donors after the first or second dose of a SARS-CoV-2 vaccine	Cwm Taf Morgannwg University Health Board,	N/A
Blood samples from convalescent patients	Cwm Taf Morgannwg University Health Board	N/A
Blood samples from healthy donors	Welsh Blood Service, Velindre NHS Trust	N/A

**Chemicals, peptides, and recombinant proteins**		

Synthetic peptide SLYNTVATL	Peptide Protein Research Ltd	N/A
Synthetic peptide ALWGPDPAAA	Peptide Protein Research Ltd	N/A
Synthetic peptide HPVGEADYFEY	Peptide Protein Research Ltd	N/A
Synthetic peptide YLQPRTFLL	Peptide Protein Research Ltd	N/A
Synthetic peptide YLQLRTFLL	Peptide Protein Research Ltd	N/A
Protein Kinase Inhibitor (Dasatinib)	Merck	Cat# SML2589
Phusion High-Fidelity DNA polymerase	Thermo Fisher Scientific	Cat# F534L
Phusion HF PCR Master Mix	Thermo Fisher Scientific	Cat# F531L
Kinase-Ligase-Dpn1 (KLD) enzyme mix	New England Biolabs	Cat# M0201L
T4 DNA Ligase	Thermo Fisher Scientific	Cat# EL0011
EcoRI restriction enzyme	Thermo Fisher Scientific	Cat# FD0274
NdeI restriction enzyme	Thermo Fisher Scientific	Cat# FD0583
XbaI restriction enzyme	Thermo Fisher Scientific	Cat# FD0684
XhoI restriction enzyme	Thermo Fisher Scientific	Cat# FD0694
Thermosensitive Alkaline phosphotase	Thermo Fisher Scientific	Cat# EF0651
Puromycin	Thermo Fisher Scientific	Cat# A1113802
Amphotericin B	Merck	Cat# A2942
Ciprofloxacin	Bayer	Cat# 40310031-3
Carbenicilin disodium salt	Thermo Fisher Scientific	Cat# 10396833
TAPI-0	Santa Cruz Biotechnology	Cat# sc-203410
Recombinant Human IL-2 (Proleukin)	Prometheus	N/A
Human IL-15	Miltenyi Biotec	Cat# 130-095-766
Sodium pyruvate	Merck	Cat# S8636
HEPES buffer	Merck	Cat# H0887
Non-Essential Amino Acids Solution	Merck	Cat# M7145
L-Phytohemagglutinin purified	Thermo Fisher Scientific	Cat# R30852701
Opti-MEM reduced serum medium	Thermo Fisher Scientific	Cat# 31985070
Polyethylenimine (PEI)	Merck	Cat# 408727
Tris(hydroxymethyl)aminomethane	Merck	Cat# 1083821000
Ethylenediaminetetraacetic acid	Merck	Cat# 324503-1KG
Triton X-100	Merck	Cat# 9036-19-5
Guanidine hydrochloride	Thermo Fisher Scientific	Cat# 24110
BirA enzyme	Avidity	Cat# BirA-RT
Isopropyl β-D-1-thiogalactopyranoside	Generon	Cat# 21530057-5
Dithiothreitol	Merck	Cat# 11583786001
Cysteamine	Merck	Cat# M9768-5G
Cystamine	Merck	Cat# C121509-25G
L-arginine	Merck	Cat# A8094-1KG
PACT Premier crystallisation screen	Molecular Dimensions	Cat# MD1-36
TOPS Screen	Bulek et al. 2012. TCR/pMHC Optimized Protein crystallization Screen. *Journal of Immunological Methods*, 382(1–2), 203–210	

**Critical commercial assays**		

RNA protect cell reagent	Qiagen	Cat# 76526
RNeasy Plus Micro kit for RNA extraction	Qiagen	Cat# 74034
SMARTer RACE 3’/5’ cDNA	Takara Clontech	Cat# 634859
Qubit dsDNA HS Assay kit	Thermo Fisher Scientific	Cat# Q32851
MiSeq Reagent Kit v2	Illumina	Cat# MS-102-2003
Anti-human CD8 magnetic microbeads	Miltenyi Biotec	Cat# 130-045-201
Q5 Site Directed Mutagenesis Kit	New England Biolabs	Cat# E0554S
Purelink Quick Plasmid Miniprep kit	Thermo Fisher Scientific	Cat# 10522723
Human MIP1-β DuoSet ELISA	R&D Systems	Cat# DY271
Anti-PE magnetic microbeads	Miltenyi Biotec	Cat# 130-048-801, RRID: AB_244373
LIVE/DEAD Fixable Violet Dead Cell Stain Kit	Thermo Fisher Scientific	Cat# L34964
Dynabeads^TM^ Human T-Activator CD3/CD28 for T Cell Expansion and Activation	Thermo Fisher Scientific	Cat# 10548353
PureLink™ HiPure Plasmid Filter Maxiprep Kit	Thermo Fisher Scientific	

**Deposited data**		

HLA A^∗^02:01-YLQPRTFLL structure	This paper	PDB:7P3D
HLA A^∗^02:01-YLQLRTFLL structure	This paper	PDB:7P3E
YLQ36:HLA A^∗^02:01-YLQPRTFLL structure	This paper	PDB:7PBE
Structure of the SARS-CoV-2 spike glycoprotein (closed state)	Walls et al., 2020	PDB: 6VXX
YLQ7:HLA A^∗^02:01-YLQPRTFLL structure	Wu et al., 2022	PDB:7N1F
NR1C:HLA A^∗^02:01-YLQPRTFLL structure	Chaurasia et al., 2021	PDB:7N6E

**Experimental models: Cell lines**		

A549 WT	Locally sourced	RRID: CVCL_0023
A549 +HLA A^∗^02:01	This paper	N/A
A549 +HLA A^∗^02:01 +Wuhan Spike	This paper	N/A
A549 +HLA A^∗^02:01 +P272L Spike	This paper	N/A
T2	Locally sourced	RRID: CVCL_2211
HEK293T	Locally sourced	RRID: CVCL_0063
CD8 T cell clone YLQ36 from patient F036	This paper	N/A
CD8 T cell clone YLQ83 from patient F014	This paper	N/A
CD8 T cell clone YLQ120 from patient F019	This paper	N/A
CD8 T cell clone YLQ132 from patient F019	This paper	N/A
Wuhan YLQPRTFLL epitope T cell line from patient M001	This paper	N/A
Wuhan YLQPRTFLL epitope T cell line from patient F014	This paper	N/A
Wuhan YLQPRTFLL epitope T cell line from patient M016	This paper	N/A
Wuhan YLQPRTFLL epitope T cell line from patient F019	This paper	N/A
Wuhan YLQPRTFLL epitope T cell line from patient F028	This paper	N/A
Wuhan YLQPRTFLL epitope T cell line from patient F030	This paper	N/A
Wuhan YLQPRTFLL epitope T cell line from patient F036	This paper	N/A
Wuhan YLQPRTFLL epitope T cell line from patient M012	This paper	N/A
Wuhan YLQPRTFLL epitope T cell line from patient F021	This paper	N/A
Wuhan YLQPRTFLL epitope T cell line from vaccinee 201a	This paper	N/A
Wuhan YLQPRTFLL epitope T cell line from vaccinee 204a	This paper	N/A
Wuhan YLQPRTFLL epitope T cell line from vaccinee 206b	This paper	N/A
Wuhan YLQPRTFLL epitope T cell line from vaccinee 208a	This paper	N/A
Wuhan YLQPRTFLL epitope T cell line from vaccinee 209b	This paper	N/A
Wuhan YLQPRTFLL epitope T cell line from vaccinee 0439	This paper	N/A

**Oligonucleotides (5’ to 3’)**		

T7 Forward primer: TAATACGACTC ACTATAGGG	Eurofins	N/A
Universal Primer A (forward): TAATACGACTCACT ATAGGGCAA GCAGTGGTATCAACGCAGAGT	Takara Clontech	Cat# 634859
TCR-Cb-R1 (reverse): GAGACCCT CAGGCGGCTGCTC	Eurofins Rius et al., 2018	N/A
TCR-Ca-R1 (reverse): CCATAGACCTCATGTCTAGCACAG	This paper, Eurofins	N/A
Barcoded ‘NEB-i50’ forward primers	This paper, Eurofins (Table S4)	N/A
Barcoded ‘NEB-i70’ reverse primers (alpha or beta TCR chain specific)	This paper, Eurofins (Table S4)	N/A
EF1a (Forward): TCAAGCCTCAGACAGTGGTTC	Eurofins	N/A
Spike seq primer 1 (p800nt): CTCTGCTCTGGAACCCCTGGT	This paper, Eurofins	N/A
Spike seq primer 2 (p1500nt): GAGTGTCTGTGATCACCCCT	This paper, Eurofins	N/A
Spike seq primer 3 (p2500nt): CGGCTTCAATTTCAGCCAGATT	This paper, Eurofins	N/A
rCD2 (Reverse): AACTTGCACCGCATATGCAT	This paper, Eurofins	N/A
Site-Directed mutagenesis primer (Forward): TGAGAACCTTCCTGCTGAAGTACAACG	This paper, Eurofins	N/A
Site-Directed mutagenesis primer (Reverse): GCTGCAGGTAGCCCACATAGTAAG	This paper, Eurofins	N/A

**Recombinant DNA**		

Transfer plasmid backbone:pELNS	pELNS plasmid kindly provided by Dr. James Riley	(University of Pennsylvania)
Transfer plasmid: pELNS HLA A2:01	This paper	N/A
Transfer plasmid backbone: pSnapFast	Oxgene	N/A
Expression plasmid: SARS-Cov2 Spike Wuhan-rCD2 pSnapFast	This paper	N/A
Expression plasmid: SARS-Cov2 Spike P272L-rCD2 pSnapFast	This paper	N/A
Envelope Plasmid: pMD2.G	Didier Trono (Addgene plasmid # 12259 ; http://n2t.net/addgene:12259)	RRID: Addgene_12259
Packaging Plasmid: pMDLg/pRRE	Didier Trono (Addgene plasmid # 12251 ; http://n2t.net/addgene:12251)	RRID: Addgene_12251
Packaging Plasmid: pRSV-Rev	Didier Trono (Addgene plasmid # 12253 ; http://n2t.net/addgene:12253)	RRID: Addgene_12253
pGEM-T expression plasmid backbone	Promega	Cat# A3600
pGEM-T truncated-HLA A^∗^02:01 (with and without a biotin tag)	Garboczi et al. 1996. HLA-A2-peptide complexes: refolding and crystallization of molecules expressed in *Escherichia coli* and complexed with single antigenic peptides. *PNAS*. 89. 3429-3433	N/A
pGEM-T Beta-2 microglobulin	Garboczi et al. 1996. HLA-A2-peptide complexes: refolding and crystallization of molecules expressed in *Escherichia coli* and complexed with single antigenic peptides. *PNAS*. 89. 3429-3433	N/A
pGEM-T YLQ36 TCR alpha chain	This paper	N/A
pGEM-T YLQ36 TCR beta chain	This paper	N/A

**Software and algorithms**		

R v4.0.5	R Core Team, 2018. https://www.R-project.org/	R Project, RRID:SCR_001905
Ggtree	Yu et al., 2017https://bioconductor.org/packages/ggtree/	ggtree, RRID:SCR_018560
Rnaturalearth	South A (2022). rnaturalearth: World Map Data from Natural Earth. https://docs.ropensci.org/rnaturalearth (website) https://cran.r-project.org/package=rnaturalearth	N/A
Tidyverse	Wickham et al., 2019https://cran.r-project.org/package=tidyverse	tidyverse, RRID:SCR_019186
Gotree	Frédéric Lemoine, Olivier Gascuel, Gotree/Goalign: toolkit and Go API to facilitate the development of phylogenetic workflows, NAR Genomics and Bioinformatics, Volume 3, Issue 3, September 2021, https://github.com/evolbioinfo/gotree	N/A
MAFFT	Katoh et al., 2002http://mafft.cbrc.jp/alignment/server/	MAFFT, RRID:SCR_011811
IQ-TREE	Nguyen et al., 2015. https://doi.org/10.1093/molbev/msu300 http://iqtree.org	IQ-TREE, RRID:SCR_017254
MixCR v3.0.13	Bolotin et al., 2015https://github.com/milaboratory/mixcr	MiXCR, RRID:SCR_018725
VDJviz	Bagaev et al., 2016. https://vdjviz.cdr3.net/	N/A
Graphpad PRISM v9.0.0	Graphpad	GraphPad Prism, RRID:SCR_002798
FlowJo	FlowJo	FlowJo, RRID:SCR_008520
Rock Maker	Formulatrix	N/A
PyMol 2.3.4	Schrodinger	PyMOL, RRID:SCR_000305
CCP4 7.1	Science and Technology Facilities Council	CCP4, RRID:SCR_007255
PHASER 2.7	Phoenix Online	PHASER, RRID:SCR_014219
Win-COOT 0.9.6	Science and Technology Facilities Council	COOT, RRID:SCR_014222
REFMAC 5.8	Science and Technology Facilities Council	REFMAC5, RRID:SCR_014225

**Other**		

FACSAria II	BD Biosciences	643178
Sony MA900 Sorter	Sony	N/A
ACEA NovoCyte 3005 with NovoSampler pro	ACEA, Agilent	N/A
BD FACSCanto II	BD Biosciences	N/A
MACSmix tube rotator	Miltenyi Biotec	Cat# 130-090-753
MiSeq	Illumina	SY-410-1003
Cellulose Nitrate Membrane Filters	Sartorius	Cat# 11306-47-N
Superdex 200 increase 10/300 size exclusion column	Cytiva	Cat# 1518208
Poros 50 HQ Anion Exchange column	Thermo Fisher Scientific	Cat# 15662135
AKTA Pure 25 L	Cytiva	Cat# 29018226
Amicon Ultra-15 Centrifuge Filter Unit	Merck	Cat# UFC901024
Crystal Gryphon Liquid Handling System	Art Robbins Instruments	Cat# 620-1000-10
Rock Imager 2	Formulatrix	N/A
PILATUS 9M pixel detector	Dectris	N/A
Streptavidin R-phycoerythrin conjugate (SAPE)	Thermo Fisher Scientific	Cat# 10104572
FcR Blocking Reagent, human	Miltenyi Biotec	Cat# 130-059-901; RRID: AB_2892112
SARS-CoV-2 Spike protein sequences	European Nucleotide Archive	Project PRJEB37886

### Resource availability

#### Lead contact

Further information and requests for resources and reagents should be directed to and will be fulfilled by the lead contact, Andrew K. Sewell (sewellak@cardiff.ac.uk). A list of critical reagents (key resources) is included in the key resources table.

#### Materials availability

Information and requests for resources and reagents may be directed to lead contact, Andrew K. Sewell (sewellak@cardiff.ac.uk).

### Experimental model and subject details

#### COVID-19 convalescent patients

All participant samples were pseudo-anonymised and the clinical Chief Investigator, Dr Lucy C Jones, was the only individual with access to the confidential record linking sample numbers to identifying information. Pseudo-anonymised sample numbers were not known to participants or to anyone outside of the research group. Our CP study was given a favourable opinion by the Health Research Authority (HRA) Research Ethics Committee London – (Brighton & Sussex), IRAS (Integrated Research Application System for HRA) 269506 and was also reviewed by Healthcare Research Wales and Cwm Taf Morgannwg University Health board, with research permissions granted.

Eligible participants were recruited during March-June 2020 and were adult (over 18) convalescent healthcare workers at Cwm Taf Morgannwg University Health Board. Inclusion criteria included a positive nasopharyngeal swab for SARS-CoV-2 by PCR, more than 28 days prior to blood sample collection. In addition to having had a positive SARS-CoV-2 swab test, all participants fulfilled the criteria for a definite diagnosis of COVID-19 at least 28 days prior to recruitment, defined by at least one of the following: new onset of pyrexia, a continuous cough, anosmia, or ageusia. Venous blood samples (50 mL) were collected from 37 informed and consented participants. This was stored in EDTA for up to 3 h and transported in UN3373 containers to the laboratory. Peripheral Blood Mononuclear Cells (PBMCs) were collected and frozen on the same day. All human blood was procured and handled in accordance with the guidelines of Cardiff University to conform to the United Kingdom Human Tissue Act 2004. HLA A^∗^02 typing was performed by flow cytometry using anti-HLA A2 antibody (BB7.2, BioLegend, San Diego, CA, US). ‘M’ and ‘F’ of the patient code denotes male or female, with 4 males (M001, M016, M012 and M029) and 9 females (F014, F019, F024, F026, F028, F030, F031 F036 and F021) used for this study. One of the patients (F031) did not respond to the YLQPRTFLL epitope from Spike glycoprotein (residues 269–277) and therefore not used for this study. Of the twelve HLA A^∗^02^+^ patients ten had mild disease and two had moderate disease in accordance with the US National Institutes of Health classification. None of the participants required treatment with invasive ventilation support. The 4 male and 8 female participants studied had ages ranging from 24 to 60 years and a median age of 48.5 years. Peripheral blood was collected between 6 and 13 weeks after the positive PCR swab (median 12 weeks).

#### SARS-CoV-2 vaccinees

We recruited six participants who had received one dose of a SARS-CoV-2 vaccine as part of the National UK vaccination programme, under ethical approval (Central University Research Ethics Committee (CUREC), University of Oxford, Approval Reference: R71346/RE001). Inclusion criteria for participants included: no clinical history of symptoms of COVID-19 since January 2020, no history of a positive COVID-19 swab or positive COVID-19 antibody test. Exclusion criteria of participants included: immunological conditions which directly affect immune responses, current or recent medications which directly affect immune responses, recent history of a transfusion of blood products and pregnancy. Peripheral blood (50 mL) was collected between 28 to 60 days after receiving one dose of a vaccine for SARS-CoV-2 and processed within 4 h of sampling. Participants received either the AstraZeneca (ChAdOx1 nCoV-19) vaccine or the Pfizer (BNT162b2 2) vaccine. Healthy donor 0439 was a colleague who received the BNT162b2 vaccine and volunteered a sample on day 14 following the second vaccine dose having heard that we were studying immune escape. Donor 0439 was the only donor to have undergone a full vaccination course at the time of study. The three male (204a, 206b and 0439) and four female (201a, 205b, 208a and 209b) donors had ages ranging from 41 to 75 years and a median age of 57 years.

#### Healthy donors

Blood samples from healthy donors were sourced from the Welsh Blood Service (Velindre NHS Trust, Wales, UK) as EDTA treated ‘buffy coats’ and ethical approval granted by the School of Medicine Research Ethics Committee (reference 18/56). Each buffy coat was seronegative for HIV-1, HBV and HCV. Blood and cells derived thereof were handled in accordance with Cardiff University guidelines in alignment with the United Kingdom Human Tissue Culture Act 2004.

#### Established cell lines

Cell lines were regularly tested for mycoplasma (MycoAlert, Lonza, Basel, Switzerland) and cultured at 37°C with 5% CO_2_ on the basis of ATCC guidelines: lung carcinoma A549 (CCL-185^TM^, male) and T2 (CRL-1992^TM^, hybrid cell line between CEMR.3 (female) and 721.174 (female)) in R10 (RPMI 1640 media supplemented with 10% heat-inactivated foetal bovine serum (FBS, (Thermo Fisher Scientific), 100 U mL^−1^ penicillin, 100 μg mL^−1^ streptomycin and 2 mM L-Glutamine, (all Merck)), immortalized embryonic kidney cell HEK293T (CRL-1573^TM^, female) in D10 (as for R10 but with DMEM (Merck)). Adherent cell lines were passaged when 50–80% confluent by detachment from tissue culture flasks with D-PBS (Merck) and 2 mM EDTA (Thermo Fisher Scientific) and split 1:10-1:20. T2 cells were cultured as suspension cells and split 1:10 twice weekly. Cell line authenticity was checked regularly; based on imagery of morphology and descriptions of growth from the ATCC (https://www.atcc.org) and cell line specific characteristics: T2 (HLA A^∗^02^low^ HLA DR^neg^ CD19^+^ and able to upregulate A2 with exogenous A2 restricted peptides), HEK293T (able to produce lentivirus) and A549 (HLA A^∗^02^neg^ and able to phagocytose bacterium to activate MAIT cells).

#### Modified cell lines

A549 cells were modified by expression of the transgenes *HLA A^∗^02*:*01*, Wuhan Spike (*S*) or P272L Spike. Modified cells were cultured in the same media as the parental cell line and maintained as enriched lines.

#### T cells

T cell clones and lines were cultured in T cell media: as for R10, supplemented with 20IU (expansion media) or 200IU (maintenance media) mL^−1^ of IL-2 (Aldesleukin, Proleukin; Prometheus), 25 ng mL^−1^ IL-15 (Miltenyi Biotec), 10 mM HEPES, 1 mM Sodium pyruvate and 1X MEM non-essential amino acids. CD8 T cell clones YLQ83, YLQ120, YLQ132 and YLQ36 specific for the HLA A^∗^02:01-restricted epitope YLQPRTFLL from Spike glycoprotein (residues 269–277) were obtained by limiting dilution in 96U well plates. T cell lines were generated by enrichment with PE conjugated tetramers and anti-PE microbeads (Miltenyi Biotec), performed *ex vivo*, or from T cells that had been previously expanded with CD3/CD28 Dynabeads (Thermo Fisher Scientific). T cells were expanded using irradiated allogeneic PBMCs from three buffy coats (Welsh Blood and 1 μg mL^−1^ Phytohaemagglutinin (PHA, Thermo Fisher Scientific).

### Method details

#### SARS-CoV-2 lineage dynamics

To characterise the distribution of mutations in the Spike epitope between positions 269–277, we performed a set of analyses making use of high quality publicly available sequence data from GISAID and COG-UK as of January 1^st^ 2022 (consortiumcontact@cogconsortium.uk, 2020).

#### Estimating *the occurance of mutations between positions 269-277*

To generate an estimate of the number of times mutations have occurred within the epitope, we performed ACCTRAN (Swofford and Maddison, 1987) ancestral state reconstruction using gotree (available at https://github.com/evolbioinfo/gotree), on a tree with 263,186 taxa, generated by COG-UK (COVID-19 Genomics UK (COG-UK), 2020), for each Spike protein site.

Subtree groups were then identified by nodes with a consensus to variant transition between positons 269–277, and this was used to infer the distribution and arisal of mutations between positions 269-277.

#### Investigation *of mutations at positon 272 and its phylogenetic and global distribution*

The phylogenetic tree for visualising variants at position 272 comprises 1227 taxa, and was generated from an alignment of consensus genome sequences using mafft version 7.475. It has previously been identified that a set of ambiguous/problematic sites exist that may impact phylogenetic inference (De Maio et al., 2021). To deal with this issue we used v4 of the problematic sites vcf (available here: https://github.com/W-L/ProblematicSites_SARS-CoV2/blob/master/archived_vcf/problematic_sites_sarsCov2.2020-12-22-11:15.vcf), and masked these from our alignment that was then used for onward phylogenetic processing using the masking tool provided through the problematic sites github repository (https://github.com/W-L/ProblematicSites_SARS-CoV2). Once the masked alignment was generated, this was used to build the phylogenetic tree using IQ-TREE version 2.1.2 (Minh et al., 2020, Nguyen et al., 2015), with a HKY + G model (Hasegawa et al., 1985), 1000 ultrafast bootstrap replicates (Hoang et al., 2018), and rooted using Wuhan isolate MN908947.3 as an outgroup. The phylogenetic trees were then drawn/visualised using ggtree (Yu et al., 2017).

To characterise the global distribution of mutations and the phylogenetic context in which mutations occurred, we collected information on case location (country, from GISAID and local authority from the COG-UK public data) and PANGO lineage for all samples with mutations at positon 272. PANGO provides a classification system that groups phylogenetically related samples, which is routinely run on all samples submitted to GISAID and/or COG-UK. The combined dataset, comprising mutations at position 272, locations and PANG lineage was then collated from GISAID and COG-UK and these were then visualised using R version 4.0.5.

#### Isolation of PBMC from whole blood

PBMCs from healthy donors used to create ‘feeder’ cells for the expansion of T cells were separated from whole blood using SepMate tubes (50 mL, Stem Cell Technologies Vancouver, Canda), whereas convalescent patient and vaccinee PMBCs were isolated using standard density centrifugation. The buffy coats from healthy donors (∼50 mL) were diluted with R10 to 150 mL and rotated overnight at 11 rpm on a digital roller shaker (SRTD6, Stuart) at RT. Each diluted buffy coat was made up to 210 mL with RPMI-1640 then placed in six SepMate tubes containing 13 mL of Histopaque 1077 (Merck) that had been warmed to room temperature, then centrifuged at 1200 x *g* for 10 min in a 5810R Eppendorf centrifuge (Hamberg, Germany), with a S-4-104 rotor, 750 mL buckets and 50 mL tube adapters. If red blood cells (RBCs) were present in the top chamber of the SepMate tubes, they were centrifuged for a further 2 min at 1200 x *g*. The upper layer from each SepMate tube was harvested, made up to 50 mL with RPMI-1640, centrifuged at 800 x *g* for 10 min, then resuspended in 25 mL of RBC lysis buffer (155 mM ammonium chloride, 10 mM potassium bicarbonate, 0.5 M EDTA, pH 7.2–7.4 using hydrochloric acid and 0.22 um filtered) and incubated for 10 min in a 37°C water bath. After lysis, 25 mL of RMPI-1640 was added and centrifuged at 300 x *g* for 5 min to remove platelets. RBC lysis was repeated if necessary. The resulting PBMC pellet was resuspended in R10 and cells counted by trypan blue exclusion. Standard density centrifugation was performed by carefully layering blood over Histopaque 1077 at a 1:1 ratio. Depending on the volume of blood being prepared, either 15 mL or 50 mL centrifuged tubes were used. The layered blood was centrifuged with no brake at 400 x *g* for 30 min. The buffy layer at the interface between the histopaque and serum was harvested using a pastuer pipette in to a 50 mL centrifuge tube and RPMI-1640 added to make the volume 50 mL. PBMCs were pelleted by centrifugation at 800 x *g* for 10 min, the supernatant discared, then to remove platelets 25 mL of RPMI-1640 before centrifugation at 300 x *g* for 5 min. The PBMC pellet was resuspended in R10 as above. No RBC lysis was used for convalescent patients or vaccinees.

#### Flow cytometry and cell sorting

Staining was performed in 5 mL polystyrene test tubes (1 wash per step) or 96U well plates (3 washes per step), using 0.5-1 x10^5^ cells per sample for T cell clones and lines, and 3-4 x10^6^ for PBMCs. Dead cells were detected with 2 μL of a 1:40 dilution of LIVE/DEAD® Fixable Violet Dead Stain Kit. Samples were acquired on a BD FACSCanto II (BD Biosciences, Franklin Lakes, NJ, USA) or ACEA NovoCyte 3005 with NovoSampler pro (ACEA Biosciences, Agilent, CA, US), then analyzed with FlowJo software (Tree Star Inc., Ashland, Oregon, US). Cells were gated on FSC-A/H versus SSC-A/H, single cells (FSC-A versus FSC-H), then viable cells (marker of choice versus LIVE/DEAD® Fixable Violet Dead Stain). Cell sorting was performed on a BD FACS Aria III (BD Biosciences) by Central Biotechnology Services at Cardiff University or on a SONY MA900 sorter (SONY Biotechnologies Inc., San Jose, CA, US). T cells for TCR sequencing were captured in RLT lysis buffer supplemented with 40 mM DTT or RNAprotect reagent (both from Qiagen). T cells or transduced A549 s were collected in their respective culture media supplemented with 25 μg mL^−1^ of Amphotericin B (Merck) and 10 μg mL^−1^ of Ciprofloxacin (Ciproxin, Bayer, Leverkusen, Germany). If samples needed to be fixed, they were incubated for 20 min on ice with 2% formaldehyde, washed again in PBS.

#### Tetramer assembly and staining

Monomeric pHLA A^∗^02:01 were generated in house as described below and used to assemble tetramers. Tetramers were assembled by 5 consecutive 20 min incubations of PE conjugated streptavidin (SAPE, Thermo Fisher Scientific) with pHLA A^∗^02:01 monomer at 1:4 molar ratio, on ice in the dark. Once assembled, 1:100 protease inhibitor (set 1, Merck, London, UK) and PBS was added to a working concentration of 0.1 mg mL^−1^ (with regards to the pHLA A^∗^02:01 component). Tetramers were stored at 4°C in the dark and used within 3 days of assembly. To remove aggregates, tetramers were centrifuged at full speed in a microfuge for 1 min immediately before staining samples. HLA A2 restricted epitopes from HIV (SLYNTVATL, p17 Gag, residues 77–85) or preproinsulin (ALWGPDPAAA, residues 15–24) were used as irrelevant tetramers, allowing gating to be set for staining with Wuhan (YLQPRTFLL) and P272L variant (YLQ**L**RTFLL) tetramers. Staining was performed by pre-treating T cell clones or lines with 50 nM of the protein kinase inhibitor, Dasatinib, at 37°C for 10–30 min in 100 μL of FACS buffer (PBS with 2% FBS), followed by the addition of 0.5 μg tetramer (with respect to pHLA A^∗^02:01 component) without washing or pre-chilling, and incubated for 30 min on ice and in the dark. Cells were then washed in PBS and stained with LIVE/DEAD® Fixable Violet Dead Stain Kit for 5 min at room temperature. Following incubation and without washing, fluorochrome-conjugated antibodies for CD3 PerCP (BW264/56, Miltenyi Biotec) and CD8 APC (REA734, Miltenyi Biotec), APC-Vio770 or FITC (both BW135/80, Miltenyi Biotec) were added and incubated for 20 min on ice in the dark. *Ex vivo* samples were also stained at the same time as the other antibodies with CD14 Pacific Blue (ME52, BioLegend) and CD19 Pacific Blue antibodies (HIB19, BioLegend). Unconjugated anti-PE (clone PE001, BioLegend) antibody was added with the other antibodies to stabilise staining with tetramer (PMID 25452566). Cells were then washed in PBS and used for flow cytometry. Gating was for lymphocytes (FSC-A/H versus SSC-A/H), single cells (FSC-A versus FSC-H), viable cells (dead stain negative), then marker(s) of interest. CD14 and CD19 positive cells were excluded for the analysis of PBMCs.

#### Generation of T cell lines and clones

T cells from patients recognising YLQPRTFLL were isolated from whole PBMCs using PE conjugated HLA A^∗^02:01-YLQPRTFLL tetramers and magnetic anti-PE beads according to the manufacturer’s guidelines (Miltenyi Biotec). The PBMCs were pre-treated with 50 nM PKI and 0.5 μg of tetramer used per 2-3 x10^6^ PBMCs, as described above. For donors M001, M012 and F021 a bulk T cell library was firstly created using magnetically purified CD8 T cells (Miltenyi Biotec) and CD3/CD28 Dynabeads according to the manufacturer’s instructions (Thermo Fisher Scientific), but with 3 beads per T cell and in T cell media containing IL-15 (T cell expansion media, as described above). After 2 weeks of expansion with CD3/CD28 beads, HLA A^∗^02:01-YLQPRTFLL PE tetramers and anti-PE beads were used as above to enrich antigen specific T cells. Tetramer enriched cells were maintained in culture overnight and then expanded with irradiated (3100 cGy) allogeneic PBMCs from buffy coats, and 1 μg mL^−1^ of PHA. Tetramer-enriched T cell lines were cloned by limiting dilution in 96 U-well plates containing 100 μL of T cell expansion media. T cells were plated at 0.5, 1 or 3 cell well^−1^with 50,000 irradiated allogeneic PBMC from 3 buffy coats and 1 μg mL^−1^ PHA. Cells were cultured for 7 days before the addition of 100 μL T cell expansion media. On day 14, wells containing visibly growing T cell clones were screened for tetramer staining or the wells restimulated with irradiated allogenic PBMCs and PHA as above. Expansion of T cell clones or T cell lines were performed in either T25 flasks or wells. For T25 flasks, up to 1 x10^6^ T cells and 15 x10^6^ irradiated allogenic PBMCs from 3 buffy coats and 1 μg mL-1 PHA in 15 mL of T cell media per flask, which was tilted at a 45° angle to allows the T cells and PBMCs to collect. On day 5, half the media was replaced with T cell expansion media and the flask kept at a 45° angle. On day 7 post-expansion, cells were washed, counted, and plated in T cell maintenance media at a density of 1.5-2 x10^6^ mL^−1^ in 48 or 24 well plates. For expansions in 24 well plates, 0.1–0.2 x10^6^ T cells were used well^−1^, with 4 x10^6^ irradiated allogenic PBMCs from 3 buffy coats and 1 μg mL^−1^ of PHA in 2 mL of expansion media. T cell grown in plates had 50% of the media changed thrice weekly. T cells were used in functional at 14–28 days post-expansion.

#### Flow cytometry based activation assays

T cells were incubated in R5 (as for R10 with 5% FBS) for 24 h before setting up the assay to help reduce spontaneous activation. For TNF-processing inhibitor (TAPI)-0 (Merck Group) assays, T cells (3 x10^4^) and target (6 x10^4^) cells were co-incubated for 4–6 h with 30 μM TAPI-0 (Merck), antibodies directed against TNF (clone cA2, Miltenyi Biotec) and CD107a (clone H4A3, BD Biosciences), with the latter detecting activation-induced degranulation of cytotoxic T cells. For peptide-pulsing, T2 cells were incubated in 1 mL of R5 with peptide for 1 h at 37°C under slow rotation using a MACSmix tube rotator (Miltenyi Biotec). Mock pulsed T2 cells were incubated with an equivalent volume of DMSO as for the peptide condition. The T2 cells were washed 3 times with 10 mL of R5, then re-counted before assay assembly. CD3/CD28 Dynabeads were used as a postivie control, with 1 μL added well^−1^. Following co-incubation, cells were washed then stained with Fixable Live/Dead Violet Dye for 5 min at RT, and without washing antibodies against CD3 PerCP (BW264/56, Miltenyi Biotec) and CD8 APC (REA734, Miltenyi Biotec) or APC Vio770 (BW135/80, Miltenyi Biotec) added and incubated for 20 min on ice. Cells were washed in PBS and acquired immediately on a flow cytometer or fixed with 2% formaldehyde for acquisition within 24 h. Gating was for lymphocytes (FSC-A/H versus SSC-A/H), single cells (FSC-A versus FSC-H), viable CD3^+^ cells (dead stain negative), CD3^+^ and CD8^+^, then displayed as TNF versus CD107a. In some assays, only the CD107a Ab was included during set-up to measure T cell degranulation.

#### Overnight T cell activation and enzyme linked immunosorbant assays (ELISA)

T cells were incubated in R5 as above. For assay, 3 ×10^4^ T cells were used in 96U well plates at 1:2 ratio with antigen-presenting cells (T2) or target cells (A549) in 100 μL of R5 medium. For peptide activation, the peptides were added directly to the assay wells without pulsing the T2 antigen-presenting cells. The peptides used were >90% purity (Peptide Protein Research, Fareham, UK) and reconstituted in DMSO at 20 mM, with subsequent dilutions made in R0 (as for R5 but with no FBS). PHA (10 μg mL^−1^) was used as a positive control. After overnight incubation 50 μL of supernatants harvested and diluted with 70 μL of R0. MIP-1β or TNF ELISAs were performed according to the manufacturer’s instructions (R&D Systems Abingdon, UK), adapted to half area Microlon high binding ELISA microplates (Greiner Bio One Limited, Stonehouse, UK) by using half the recommended volume of reagents and buffers. All assayed samples were performed in duplicate.

#### Lentivirus production and transduction

Codon-optimised Wuhan Spike (*S*), P272L Spike (site directed mutagenesis of Wuhan Spike, details below) and *HLA A^∗^02*:*01* genes were synthesised (GeneArt, ThermoFisher Scientific) and cloned into a 3^rd^ generation lentiviral plasmids. Spike genes were designed with a rCD2 co-marker: XbaI-Kozak-Spike-XhoI-P2A-rCD2-SalI-Stop and cloned in to pSnapFast (pSF) (Oxgene, Oxford Genetics Ltd, Littlemore, Oxford, UK). The pSF plasmid was modified by removal of a XhoI site present in the plasmid backbone and insertion of stop codons after the SalI site. *HLA A^∗^02*:*01* was cloned in to pELNS (Kind gift from James Riley): XbaI-Kozak-HLA A2-Stop-XhoI. Genes were cloned by restriction digestion using XbaI, XhoI, and Thermosensitive Alkaline Phosphatase (TAP, all Thermo Fisher Scientific) and following the manufacturer’s guidelines. Ligation of the genes into pSF or pELNS was completed with T4 DNA Ligase (Thermo Fisher Scientific) and the plasmids were transformed using the heat shock method into XL10-Gold Ultracompetent cells (XL10-Gold, Agilent Technologies Santa Clara, CA, US) as per the manufacturer’s guidelines. To achieve sufficient DNA for transfection XL10-Gold cells transformed with plasmid DNA were grown in 200 mL LB media (10 g L^−1^ Typtone, 10 g L^−1^ Sodium Chloride 5 g L^−1^ yeast extract all Merck) and the DNA was extracted from the cells using the PureLink™ HiPure Plasmid Filter Maxiprep Kit (Thermo Fisher Scientific) as per the manufacturer’s protocol. For transfection, pSF or pELNS (1.52 μg), envelope plasmid (pMD2.G; 0.72 μg) and packaging plasmids (pMDLg/pRRE; 1.83 μg and pRSV-REV; 1.83 μg) were mixed in 300 μL of Opti-MEM, reduced serum medium (Thermo Fisher Scientific) followed by mixing with 1 μg μL^−1^ Polyethylenimine (PEI; Merck Group) at a 3:1 PEI: plasmid ratio. Plasmid/PEI mixtures were incubated at room temperature for 15 min, added dropwise to the HEK293T cells (80% confluence in one well of a 6-well plate) and incubated at 37°C in a 5% CO_2_ humidified atmosphere. The supernatants containing lentiviral vectors were harvested, 0.4 μm filtered, and used immediately for transduction or stored at −80°C and only defrosted once before transduction. A549 cells were transduced by spinfection with viral supernatant at 400 x *g* for 2 h, incubated at 37°C overnight and media was replaced the following morning. HLA A^∗^02:01 transduction was confirmed by staining with BB7.2 Ab. Spike transduced cells were firstly checked for with conjugated anti-rCD2 antibody (OX-34, BioLegend), then in follow-up experiments with 1 μg test^−1^ of unconjugated anti-SARS Spike glycoprotein antibody (1A9, Abcam, Cambridge, MA, US). An unconjugated isotype matched antibody (P3.6.2.8.1, eBiosciences, Thermo Fisher Scientific) was used as a control. A goat anti-mouse secondary antibody (multiple adsorbed PE conjugated Ig polyclonal; BD Biosciences, Oxford, UK) was used to detect Spike antibody staining, with 0.1 μg used stain^−1^.

#### Site directed mutagenesis of the Wuhan Spike gene

Oligonucleotides were designed to substitute the Proline to a Leucine at position 272. Non-overlapping sequences were designed so that the 5’ end of the forward oligo (5' TGAGAACCTTCCTGCTGAAGTACAACG 3’) started with the nucleotides to be mutated and included at least 20 complementary nucleotides on the 3’ side of the mutation. The reverse oligo (5' GCTGCAGGTAGCCCACATAGTAAG 3') was designed in the other direction so that the 5’ end annealed back-to-back with the forward oligo. Oligonucleotides were purchased from Eurofins Genomics (Eurofins Genomics Germany GmbH). PCR was performed using Phusion HF PCR Master Mix (Thermo Fisher Scientific). Cycling conditions were performed according to manufacturer instructions, except elongation which was done at 1 min kb^−1^. PCR products sizes were verified by running samples on 1% agarose gel. Correctly amplified products were treated by Kinase-Ligase-Dpn1 (KLD) enzyme mix (New England Biolabs, Ipswich, MA, USA) for 30 min at room temperature. Samples were then transformed into XL10-Gold Ultracompetent cells (Agilent Technologies) by heat shock and cells were plated on LB-Agar (LB media with 15 g L^−1^ Agar, Thermo Fisher Scientific) plate with Carbenicillin antibiotic (50 mg L^−1^; Thermo Fisher Scientific). Plasmid DNA was extracted from antibiotic resistant colonies using a HiPure Miniprep kit (Thermo Fisher Scientific). DNA samples were sent to Eurofins Genomics for Sanger sequencing using the following primers: EF1a forward: 5’ TCAAGCCTCAGACAGTGGTTC 3’, Spike seq primer 1(p800nt): 5'CTCTGCTCTGGAACCCCTGGT3’, Spike seq primer 2 (p1500nt): 5'GAGTGTCTGTGATCACCCCT3', Spike seq primer 3 (p2500nt): 5'CGGCTTCAATTTCAGCCAGATT3', Spike seq primer 4 (p2800nt): 5'CAACAGCGCCATCGGCAAGA3' and rCD2 reverse 5’AACTTGCACCGCATATGCAT3’.

#### HLA A2 peptide binding assay

T2 cells were plated in a 96U-well plate (2 ×10^4^ per well) in serum-free media. Wuhan (YLQPRTFLL) and P272L variant (YLQLRTFLL) peptides (>90% purity, from Peptide Protein Research) were added at 100 μM concentrations; alongside a negative control peptide HPVGEADYFEY from Epstein Barr Virus (EBNA1, residues 323–450) that binds HLA B^∗^35:01. The percentage volume of DMSO was matched in each condition. Cells were then incubated overnight at room temperature. The following day, cells were stained with anti-HLA A2 antibody (clone BB7.2, BioLegend) and incubated at 37°C for 1 h. Cells were then washed in PBS and stained with LIVE/DEAD™ fixable violet dead stain (Thermo Fisher Scientific) and analysed using flow cytometry.

#### TCR sequencing

A minimum of 5 ×10^4^ T cells were harvested from culture, washed in PBS and resuspended in 300 μL of RNAprotect cell reagent (Qiagen, Hilden, Germany). Flow sorted populations of T cells were captured directly in to RNAprotect cell reagent. RNA extraction was carried out using the RNEasy Micro kit (Qiagen). cDNA was synthesized using the 5′/3′ SMARTer kit according to the manufacturer’s instructions (Takara Bio, Paris, France). The SMARTer approach used a Murine Moloney Leukaemia Virus (MMLV) reverse transcriptase, a 3′ oligo-dT primer and a 5′ oligonucleotide to generate cDNA templates flanked by a known, universal anchor sequence at the 5′. A Step-Out PCR was performed using a pair of primers consisting of 3’ TRAC or TRBC-specific reverse primer (Eurofins Genomics) and a 5’ universal anchor-specific forward primer (Takara Bio, Paris, France). All samples were used for the following PCR reaction: 2.5 μL template cDNA, 0. 5 μL High Fidelity Phusion Taq polymerase, 10 μL 5X Phusion buffer, 0.5 μL DMSO, 1 μL dNTP Mix (stock concentration of 10 mM of each) (all from Thermo Fisher Scientific), 1 μL of TRAC or TRBC-specific primer (10 μM stock), 5 μL of 10X anchor-specific universal primer (Takara Bio, Paris, France), and nuclease-free water for a final reaction volume of 50 μL (cycling conditions: 5 min at 94°C, 30 cycles of 30 s at 94°C, 30 s at 63°C for alpha chains or 30 s at 66°C for beta chains, 120 s at 72°C). Subsequently, 5 μL of the Step-out PCR products were taken for a nested PCR, using 1 μL of barcoded forward (universal) and reverse (TRAC or TRBC) primers (10 μM stock) (Eurofins Genomics) (Table S4), 0.5 μL High Fidelity Phusion Taq polymerase, 10 μL 5X Phusion buffer, 0.5 μL DMSO, 1 μL dNTP Mix (stock concentration of 10 mM each) and nuclease-free water for a final reaction volume of 50 μL (cycling conditions: 5 min at 94°C, 30 cycles of 30 s at 94°C, 30 s at 62°C, 120 s at 72°C, and a final 10 min at 72°C). The final PCR products were loaded on a 1% agarose gel and purified with the Monarch® gel extraction kit (New England Biolabs). Purified products were sequenced on an Illumina MiSeq instrument using the MiSeq v2 reagent kit (Illumina, Cambridge, UK) according to the manufacturer’s instructions. Sequence analysis was performed using MiXCR software (v3.0.7) (Bolotin et al., 2015). Public TCR clonotypes were identified using the VDJdb database (Shugay et al., 2018).

#### Soluble inclusion body production

Synthetic genes for *HLA A^∗^02*:*01*, *Beta-2-microglobulin* (*B2M)*, YLQ36 α chain and YLQ36 β chain were designed and codon optimized for *Escherichia coli* expression, purchased from GeneArt and cloned into the pGEM-T expression vector (Promega). Designs took the form of NdeI-Gene-Stop-Stop- EcoRI and were cloned by double digestion at 37°C with NdeI and EcoRI restriction enzymes (Thermo Fisher Scientific) for 30 min. The pGEMT vector was simultaneously digested under the same conditions with NdeI, EcoRI, and TAP (all Thermo Fisher Scientific), the reaction was stopped by heating at 65°C for 5 min. Genes were ligated into the pGEM-T vector using T4 DNA ligase and following the manufactures guidelines. Sanger DNA sequencing (Eurofins Genomics) was used to confirm correct DNA sequences. TCR genes were designed to contain a non-native disulphide bond, by substituting residues 48 and 57 of the YLQ36 α chain and YLQ36 β chain constant domains respectively with cysteine residues in order to produce stable soluble TCRs with the “Boulter disulphide” bond (Boulter et al., 2003). The expressed protein sequence is shown in Figure S6. A biotin tag (GLNDIFEAQKIEWHE) was added to the C-terminus of the HLA A^∗^02:01 heavy chain construct to produce biotinylated pMHC monomers. Genes in the pGEM-T vector were transformed into competent BL21 DE3 *E*. *coli* cells (Thermo Fisher Scientific) and plated onto Luria Broth (LB) agar plates (10 g L^−1^ tryptone (Merck), 5 g L^−1^ yeast extract (Merck), 10 g L^−1^ sodium chloride (ThermoFisher Scientific), 15 g L^−1^ bacteriological agar (Oxoid)) containing 50 μg mL^−1^ of carbenicillin (ThermoFisher Scientific). Successful colonies were used to inoculate 20 mL of TYP media (16 g L^−1^ tryptone, 16 g L^−1^ yeast extract, 5 g L^−1^ sodium chloride, 3.3 g L^−1^ potassium phosphate dibasic (Merck)), which was incubated at 37°C, 220 RPM overnight to produce starter cultures. The starter cultures were then used to inoculate 1 L of TYP media, which was incubated at 37°C, 220 RPM until the OD_450_ measured 0.5. 500 μL of 1M Isopropyl β-D-1-thiogalactopyranoside (IPTG, Neo Biotech, France) was then added to the cultures to induce protein expression, before incubation for 3 h at 37°C, 220 RPM. The cultures were centrifuged for 20 min at 2786 *g* (Eppendorf 5810R) before re-suspending the subsequent pellet in 40 mL of lysis buffer (10 mM tris(hydroxymethyl)aminomethane (TRIS) pH 8.1 (Merck), 10 mM magnesium chloride, 150 mM sodium chloride, 10% v v^−1^ glycerol (Thermo Fisher) Scientific). The resuspended cultures were then freeze-thawed and sonicated at 50% power with a 2 × 10% pulse for 20 min using a Bandelin SONOPULS Ultrasonic H sonicator with a Z659169 sonicator tip (Bandelin, Berlin, Germany). 100 μg mL^−1^ of DNase (Merck) was then added before incubation at 37°C for 2 h. The suspension was diluted with 200 mL Triton wash buffer, 50 mM TRIS pH 8.1, 100 mM sodium chloride, 2 mM Ethylenediaminetetraacetic acid (EDTA, Merck), 0.5% v v^−1^ Triton X-100 (Merck)) then centrifuged for 20 min at 17,700 x *g* (Beckman Avanti JE with JA 10 rotor and 500 mL polypropylene bottle w/cap assembly). The resulting pellet was resuspended in another 200 mL of triton wash and the centrifugation step repeated. The pellet was then resuspended in 200 mL of resuspension buffer (50 mM TRIS pH 8.1, 100 mM sodium chloride, 2 mM EDTA) and centrifuged for 20 min at 17,700 x *g*. The resulting pellet was resuspended in 20 mL of guanidine buffer (50 mM TRIS pH 8.1, 100 mM sodium chloride 2 mM EDTA, 6 M guanidine hydrochloride (Thermo Fisher Scientific)), resulting in soluble inclusion bodies.

#### Soluble protein refolding

Soluble inclusion bodies were refolded by dilution of denaturing conditions to produce soluble TCR and peptide-MHC molecules (Laugel et al., 2005). Soluble peptide-MHC molecules were refolded by incubating 30 mg of HLA A^∗^02:01 heavy chain, 30 mg of B2M, 4 mg of peptide and 10 mM dithiothreitol (DTT, Merck) together at 37°C for 30 min. After incubation, the mixture of heavy chain, B2M and peptide was then added to the 1 L of peptide-MHC refold buffer (0.74 g of cysteamine (Merck) and 0.83 g of cystamine (Merck) 50 mM TRIS pH 8.1, 2 mM EDTA, 400 mM L-Arginine (Merck)). The pMHC refold buffer was continually stirred at 4°C for 6 h. Soluble YLQ36 TCR molecules were refolded by incubating 30 mg of YLQ36 TCR α chain inclusion bodies with 10 mM DTT at 37°C for 30 min 30 mg of YLQ36 TCR β chain inclusion bodies were also incubated with 10 mM DTT for 30 min, with incubation beginning 15 min after the TCRα chain incubation. 0.74 g of cysteamine and 0.83 g of cystamine were added to 1 L of TCR refold buffer (50 mM TRIS pH 8.1, 2 mM EDTA, 2.5 M urea (Merck)) at 4°C whilst the TCR chains were incubating at 37°C. After incubation the TCR chains are added to the 1 L of TCR refold buffer, with the α chain added 15 min before the β chain. The TCR refold buffer was continually stirred at 4°C for 6 h. After the 6 h incubation of TCR or peptide-MHC refold buffers, the buffer was dialysed (Cellulose dialysis membrane, Merck) in 10 mM TRIS pH8 buffer until the conductivity measured below 1 mS cm^−1^.Mixtures were then filtered through a 0.45 μm cellulose nitrate filter (Sartorius AG, Göttingen, Germany) in preparation for purification (Laugel et al., 2005).

#### Protein purification

All protein purification steps were carried out using a AKTA Pure Fast Protein Liquid Chromatography (FPLC) machine (Cytiva) following manufacturers guidelines. Proteins refolded via the standard refold protocol were dialysed into 10 mM TRIS and subjected to anion exchange purification using a POROS HQ50 column (Thermo Fisher Scientific). Protein was bound to the column using a flow rate of 20 mL min^−1^, before gradient elution with 10 mM TRIS, 1 M sodium chloride buffer at 5 mL min^−1^ in 1 mL fractions. Fractions containing the protein of interest, as determined by A_280_ were combined and concentrated to >1 mL using Amicon Ultra 10,000 kDa spin columns (Merck). Proteins purified via anion exchange were then purified by Size Exclusion Chromatography (SEC) using a Superdex 200/15 GL column (Cytiva). Proteins were injected down the column at 0.5 mL min^−1^ and eluted in 1 mL. The buffer used depended on the downstream experiments; PBS was used for use in tetramer staining and 10 mM TRIS, 10 mM sodium chloride buffer was used for crystallography. Fractions containing the protein if interest were retained for downstream experiments.

#### Peptide:MHC monomer biotinylation

After anion exchange, soluble pMHC protein containing a biotin tag was concentrated to 450 μL using Amicon 10,000 kDa Millipore spin columns. 1 μL of 1 mg mL^−1^ BirA enzyme (Avidity LLC, Auroro, CO, USA) and 50 μL of 10X SuperMix (Avidity) containing 55 mg mL^−1^ ATP (Avidity) were added to the soluble pMHC, that was then incubated overnight at room temperature. The excess biotin was then washed out by buffer exchanging into PBS using Amicon ultra 10,000 kDa spin columns (Merck) and the refold proceeded to size exclusion chromatography purification.

#### Sitting-drop crystallisation

TCR and pMHC protein crystals were grown at 18°C by sitting drop vapour diffusion using the following protocol. Directly after size exclusion chromatography TCR or pMHC at a concentration of 10 mg mL^−1^, in a buffer of 10 mM Tris and 10 mM sodium chloride buffer, was dispensed in 200 nL drops into 3 well low profile Intelliplates (Art Robbins Instruments LLC, CA, USA) using the Gryphon liquid handling robot (Art Robins Instruments). Potential crystallisation conditions were created by supplementing the protein with 200 nL from a 96 well crystallisation screen and loading the reservoir with 60 μL of the same condition. For crystallisation of TCR:pMHC complexes, both components were mixed in a 1:1 molar ratio after size exclusion chromatography and followed the same process as described above. The PACT screen (Molecular Dimensions, Anatrace Products, LLC, OH, USA) (Newman et al., 2005) and T cell optimised crystal screen (Bulek et al., 2012) were used for this study. The conditions used for each structure are reported in the statistics table (Table S2). The plates were sealed using ClearVue adhesive film sheets (Molecular Dimensions) and stored at 18°C. Plates were periodically imaged using Rock Imager 2 and Rock Maker (both Formulatrix, MA, USA).

#### Protein crystal seeding

HLA A^∗^02:01-YLQPRTFLL protein crystals were grown at 18°C via seeding. HLA A^∗^02:01-YLQLRTFLL protein crystals grown in 0.2 M sodium bromide, 0.1 M Bis Tris propane pH 8.5, 20% w v^−1^ PEG 3350 were diluted in mother liquor solution and crushed into sub-microscopic fragments using a seeding kit (Qiagen LTD, Maryland) to form crystal seed. HLA A^∗^02:01-YLQPRTFLL protein crystals were set via hanging drop by mixing 1 μL of protein sample, 0.5 μL of crystal seed, and 1 μL of crystallisation buffer (Table S2) in a well by hand, before suspending above a reservoir containing 500 μL of crystallisation buffer.

#### 3D structure determination and analysis

The reservoir solution of the successful crystallisation condition was supplemented with 20% ethyleneglycol (Molecular Dimensions) 1 μL of supplemented reservoir solution was added to the drop containing the crystals to provide cryoprotection. A magnetic ActiLoop (Molecular Dimensions) was used to harvest the crystals from the drop, prior to storage in liquid nitrogen. The collected crystals were then sent to the Diamond Light Source synchrotron in Oxfordshire, UK. X-ray datasets were collected using a PILATUS 9M pixel detector at wavelength of 0.98 Å and consisted of 3600 images, with 0.1° oscillation and 0.1 s exposure at the I04-1 MX beamline at the Diamond Light Source. Datasets were processed using the DIALS, xia2, XDS, and AUTOPROC pipelines to produce reflection intensities and to determine unit cell dimensions and symmetry space group. The CCP4 version 7.1 software suit was used to derive 3D models from the reflection intensities (Winn et al., 2011) (Collaborative Computational Project No. 4, Didcot, UK). Phaser version 2.7 was used to conduct molecular replacement, producing a 3D structure solution (McCoy et al., 2007). Win-Coot version 0.9.6 was used to adjust the amino acid sequence after molecular replacement and add relevant solvents to the 3D structure (Emsley and Cowtan, 2004). REFMAC version 5.8 was then used to refine the 3D structure (Murshudov et al., 2011). Graphical representations were prepared with the PYMOL molecular graphics system, version 2.3.4 (Schrodinger, LLC). The reflection data and final coordinates were deposited in the PDB database www.rcsb.org (HLA A^∗^02:01-YLQPRTFLL PDB: 7P3D and HLA A^∗^02:01-YLQLRTFLL PDB: 7P3E, YLQ36-HLA A^∗^02:01-YLQPRTFLL PDB: 7PBE).

#### Data display

Unless stated otherwise, all data were displayed using GraphPad Prism software. TCR V-J usage plots were generated using VDJ tools (Murshudov et al., 2011).

### Quantification and statistical analysis

For peptide sensitivity assays the EC_50_ of activation was calculated using GraphPad Prism. Bar graphs of tetramer staining or T cell activation and display the mean of duplicate values with error bars dipicitng the SEM.

## Data Availability

SARS-CoV-2 Spike protein sequences are deposited at the European Nucleotide Archive under project PRJEB37886.

## Supplemental information

Table S1. A breakdown of numbers of sequenced cases possessing P272L in public databases, as determined by outbreak.info, downloaded on the 31st of January 2022, related to Figures 1 and 2 The number of sequenced cases in public databases possessing P272L is broken down by PANGO lineage. B.1.177 and its sublineages are displayed below the other lineages where the mutation is found to simplify reading of the table. Lineages that have WHO variant designations are highlighted in dark grey (variants of concern) and mid grey (variants of interest)

Table S2. Data collection and refinement statistics, related to Figure 7 HLA A^∗^02:01 allele, presenting YLQPRTFLL and escape mutant YLQLRTFLL and the complex of YLQ36 TCR: HLA A^∗^02:01-YLQPRTFLL. ^∗^ One crystal was used for determining each structure. ^∗∗^ The resolution range used in the final refinement of the complex structure was trimmed to 3.0–113.83 Å due to tNCS. ^1^ Coordinate Estimated Standard Uncertainty in (Å), calculated based on maximum likelihood statistics

Table S3. Contacts between the YLQ.36 TCR and HLA A∗02:01-YLQPRTFLL, related to Figure 7

Table S4. Oligonucleotides used for next generation sequencing of TCR chains, related to STAR methods
